# Mediator MED23 cooperates with RUNX2 to drive osteoblast differentiation and bone development

**DOI:** 10.1038/ncomms11149

**Published:** 2016-04-01

**Authors:** Zhen Liu, Xiao Yao, Guang Yan, YiChi Xu, Jun Yan, Weiguo Zou, Gang Wang

**Affiliations:** 1 State Key Laboratory of Cell Biology, CAS Center for Excellence in Molecular Cell Science, Institute of Biochemistry and Cell Biology, Shanghai Institutes for Biological Sciences, Chinese Academy of Sciences, 320 Yueyang Road, Shanghai 200031, China; 2 CAS-MAP Partner Institute for Computational Biology, Shanghai Institutes for Biological Sciences, Chinese Academy of Sciences, 320 Yueyang Road, Shanghai 200031, China

## Abstract

How lineage specifiers are regulated during development is an outstanding question, and the molecular regulation of osteogenic factor RUNX2 remains to be fully understood. Here we report that the Mediator subunit MED23 cooperates with RUNX2 to regulate osteoblast differentiation and bone development. *Med23* deletion in mesenchymal stem cells or osteoblast precursors results in multiple bone defects similar to those observed in *Runx2*^+/−^ mice. *In vitro*, *Med23*-deficient progenitor cells are refractory to osteoblast differentiation, and *Med23* deficiency reduces *Runx2*-target gene activity without changing Runx2 expression. Mechanistically, MED23 binds to RUNX2 and modulates its transcriptional activity. Moreover, *Med23* deficiency in osteoprogenitor cells exacerbates the skeletal abnormalities observed in *Runx2*^*+/*−^ mice. Collectively, our results establish a genetic and physical interaction between RUNX2 and MED23, suggesting that MED23 constitutes a molecular node in the regulatory network of anabolic bone formation and related diseases.

## Abstract (web summary)

The transcription factor Runx2 regulates osteoblast differentiation of mesenchymal stem cells. In this manuscript, the authors, using a specific conditional knock-out mouse model and molecular studies, demonstrate that the mediator subunit MED23 binds to Runx2 and is essential for driving mesenchymal stem cells toward an osteoblast fate.

## Graphical abstract

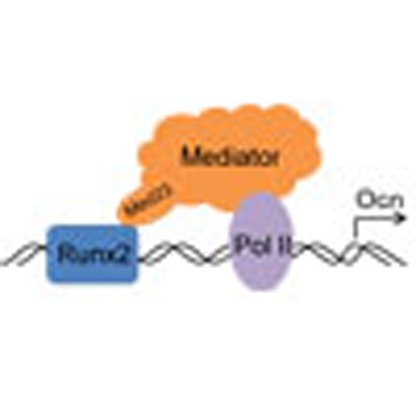

Mesenchymal stem cells (MSCs) are multipotent progenitors that can self-renew and differentiate into several distinct cell lineages, including osteoblasts, chondrocytes and adipocytes^1^,^2^. Their differential potentials and intrinsic properties make MSCs of great interest for applications in a variety of cell-based therapies^3^,^4^,^5^,^6^. In the past few decades, several master regulators that specify the various cell fates associated with MSCs have been identified: for example, *Runx2* triggers MSCs to differentiate into osteoblasts^7^,^8^. The function of *Runx2* in osteoblast differentiation was initially demonstrated by observations that *Runx2*^−/−^ mice lack both mature osteoblasts and a mineralized skeleton^8^,^9^,^10^. Although *Runx2*^+/−^ mice do undergo skeletal mineralization, the process is impaired, with delayed closure of the fontanelles and clavicular hypoplasia^10^, resembling the cleidocranial dysplasia (CCD) phenotype that arises from *Runx2* haploinsufficiency in humans^9^. The skeletal pathologies in humans and mice highlight the importance of the precise regulation of *Runx2* activity during osteogenesis.

Apart from these key transcription factors, several cofactors have been found to be involved in the fine tuning of cell-fate determination^11^,^12^,^13^. One such cofactor is the transcriptional complex Mediator, which is a multiprotein complex that has been evolutionarily conserved from yeast to metazoans. The Mediator complex is recruited by transcription factors in response to developmental or environmental signals. Through direct protein interactions, Mediator links transcription factors to the RNA polymerase II (Pol II) machinery and modulates the transcriptional output^14^.

Our previous research demonstrated that the Mediator MED23 subunit acts as a molecular modulator to control the balance between adipogenesis and myogenesis^15^,^16^. However, it remained unknown whether MED23 is also involved in the development of osteoblasts, which, like adipocytes and myocytes, are also derived from mesenchymal progenitors. In this study, we provide evidence of a role for MED23 in osteogenesis. Mice deficient in *Med23* in MSCs or preosteoblasts display defective bone formation and impaired osteoblast differentiation, resembling the skeletal phenotype of *Runx2*^+/−^ mice. Further molecular studies demonstrate that MED23 cooperatively interacts with RUNX2 in the regulation of osteogenesis. Our results reveal that MED23 is an important regulator of osteogenesis and is essential for the transcriptional activity of RUNX2.

## Results

### Med23 deficiency in MSCs impaired bone ossification

**Figure 1:**
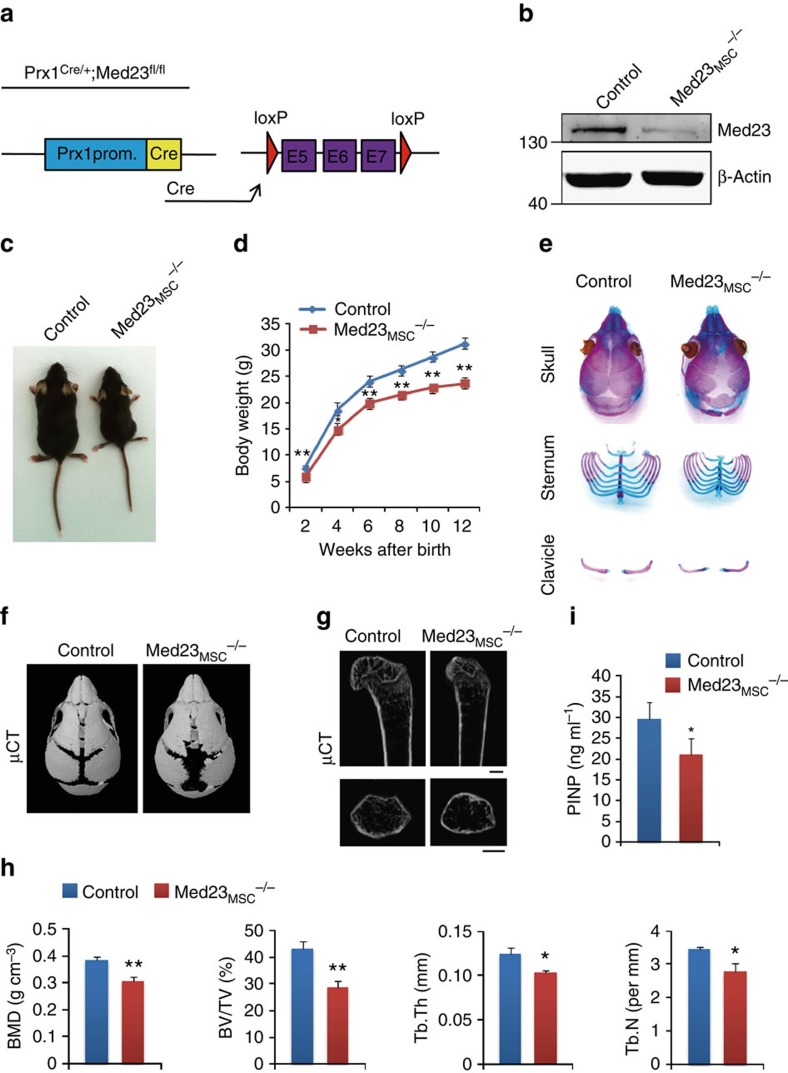
Inactivation of *Med23* in mesenchymal stem cells causes impaired bone ossification. (**a**) Illustration of *Med23* deletion in Prx1-expressing mesenchymal stem cells. (**b**) Expression of *Med23* protein in bone marrow-derived mesenchymal stem cells from 6- to 8-week-old control (*Med23*^fl/fl^) and *Med23*_MSC_−/− mice. (**c**) Representative view of 1-month-old control and *Med23*_MSC_^−/−^ mice. (**d**) Body weight of male control mice and *Med23*_MSC_^−/−^ littermates measured at different age points (*n*=3 for each group, *t*-test). Data represent means±s.d. *P<0.05, **P<0.01. (**e**) Skeletal preparations from control and *Med23*_MSC_^−/−^ newborns were double stained with Alizarin red and Alcian blue. (**f**) Microcomputed tomography (μCT) image of skulls from control and *Med23*_MSC_^−/−^ mice at postnatal day 6 (P6). (**g**) μCT images of distal femurs from 1-month-old control and *Med23*_MSC_^−/−^ mice (top panel, longitudinal view; bottom panel, axial view of the metaphyseal region). Scale bar, 1 mm. (**h**) Quantitative μCT analysis of distal femurs from 1-month-old control (*n*=5) and *Med23*_MSC_^−/−^ mice (*n*=4), including bone mass density (BMD), bone volume per tissue volume (BV/TV), trabecular thickness (Tb.Th) and trabecular number (Tb.N). Data represent means±s.e.m. *t*-test,*P<0.05, **P<0.01. (**i**) ELISA analysis of serum PINP (ng ml^−1^) from 1-month-old control and *Med23*_MSC_^−/−^ mice (*n*=4). Data represent means±s.d. *t*-test, *P<0.05, **P<0.01.

To investigate the role of *Med23* in the commitment of MSCs to an osteoblast fate, we generated a *Med23* conditional knockout mouse model by crossing *Med23*^*fl/fl*^ mice^17^ with *Prx1-Cre* mice (Fig. 1a). The latter is a transgenic line wherein Cre activity is largely confined to ventral and craniofacial mesenchyme^18^,^19^. Western blot assays verified that MED23 was largely abrogated in bone marrow-derived MSCs (Fig. 1b). MSC-specific *Med23* deletion had no effect on survival or fertility. However, *Med23*^*fl/fl*^; *Prx1-Cre* mice (*Med23*_*MSC*_^*−/−*^) were shorter and underweight relative to their control littermates. Furthermore, as the *Med23*_*MSC*_^*−/−*^ mice aged, they developed a marked dwarfism phenotype with short legs that was independent of sex (Fig. 1c,d). The heterozygous mice (*Med23*_MSC_^+/−^) appear normal (Supplementary Fig. 1d). Consistent with this observation, the level of MED23 was barely reduced in bone tissue samples from *Med23*_MSC_^+/−^ mice (Supplementary Fig. 1c).

Notably, the membranous ossification of the skull was impaired in *Med23*_*MSC*_^*−/−*^ newborns compared with control littermates. The anterior and posterior fontanelles were enlarged and the cranial sutures were wider in *Med23*_*MSC*_^*−/−*^mice. Defective osteogenesis was not limited to cranial tissues, as the clavicles became thinner and shorter, and the sternum xiphoid was also hypoplastic (Fig. 1e). And these defects occurred as early as E15.5 when the bone mass is formed (Supplementary Fig. 1a). By contrast, a skeletal analysis of *Med23*_*MSC*_*+/*− mice confirmed that they were phenotypically normal relative to control littermates (Supplementary Fig. 1b).

To further determine the function of MED23 in the skeletal system, we utilized micro-quantitative computed tomography micro computed tomography (μ-CT) to compare skeletal elements isolated from *Med23*_*MSC*_^*−/−*^ mice with corresponding elements from control littermates. As shown in Fig. 1f, 6-day-old *Med23*_*MSC*_^*−/−*^ mice displayed hypomineralization of the calvaria compared with control mice. In addition, we found that 1-month-old *Med23*_*MSC*_^*−/−*^ mice were osteopenic, with reduced bone mineral density (BMD) and bone volume per tissue volume) (BV/TV) in the femoral trabecular bone relative to age-matched control littermates. Further analysis showed that the reduced trabecular number (Tb.N) of *Med23*_*MSC*_^*−/−*^ mice was accompanied by decreased trabecular thickness (Tb.Th) and a reduction in the Tb.N compared with those of control mice (Fig. 1g,h). Consistent with the decreased BMD in *Med23*_*MSC*_^*−/−*^ mice, an ELISA assay of PINP (N-terminal propeptide of type I procollagen), a marker of bone formation, revealed a reduced bone formation rate in *Med23*_*MSC*_^*−/−*^ mice (Fig. 1i). In adults, bone is continuously remodelled by bone-resorbing osteoclasts and bone-forming osteoblasts^20^. We performed a histological analysis and *in vitro* osteoclastogenesis experiment to exclude the possibility that the decreased bone density in the long bones of *Med23*_*MSC*_^*−/−*^ mice could be attributed to increased osteoclast differentiation and/or activity. We found that osteoclast activity was comparable between *Med23*_*MSC*_^*−/−*^ mice and their control littermates (Supplementary Fig. 2a,b). Moreover, the expression of osteoclastic genes did not change either (Supplementary Fig. 2c). Taken together, these results suggest an important role for *Med 23* in bone formation.

### Ablation of *Med23* in preosteoblasts reduced bone formation

**Figure 2:**
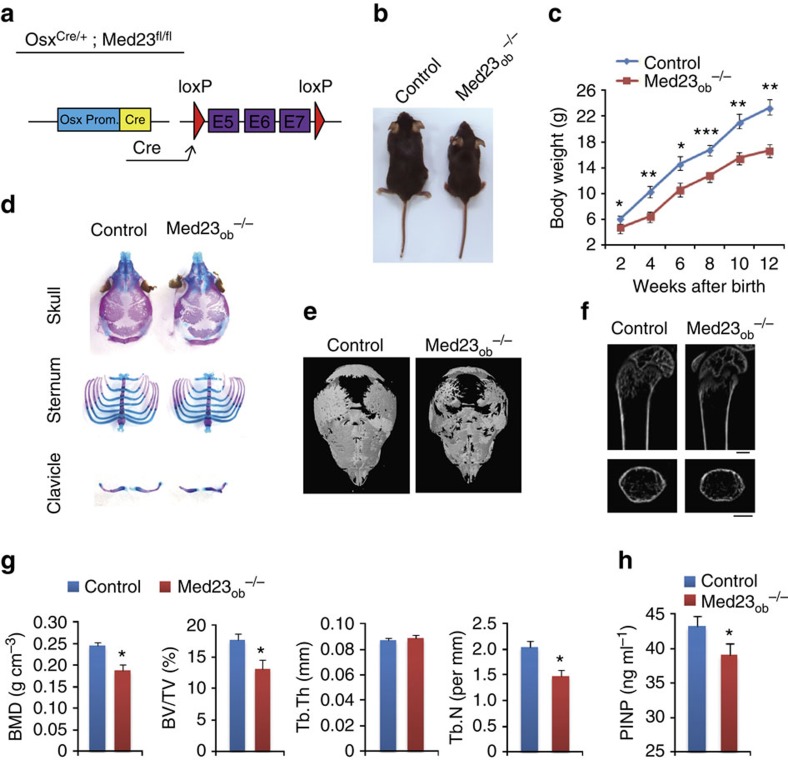
Inactivation of *Med23* in osteoblast progenitors results in defects in bone formation. (**a**) Illustration of *Med23* deletion in *Osx*-expressing osteoblast progenitors. (**b**) Representative view of 1-month-old control (*Med23*_ob_^+/−^) and *Med23*_ob_^−/−^ mice. (**c**) Body weight of male control mice and *Med23*_ob_^−/−^ littermates measured at different age points (*n*=3). Data represent means±s.d. *t*-test, *P<0.05, **P<0.01, ***P<0.001. (**d**) Skeletal preparations from control and *Med23*_ob_^−/−^ newborns were double stained with Alizarin red and Alcian blue. (**e**) μCT image of skulls from control and *Med23*_ob_^−/−^ mice at postnatal day 6 (P6). (**f**) μCT images of distal femurs from 1-month-old control and *Med23*_ob_^−/−^ mice. Scale bar, 1 mm. (**g**) Quantitative μCT analysis of trabecular bone in the distal femurs from 1-month-old control and *Med23*_ob_^−/−^ mice (*n*=3). Data represent means±s.e.m. *t*-test, *P<0.05. (**h**) ELISA analysis of serum PINP (ng ml^−1^) from 1-month-old control and *Med23*_ob_^−/−^ mice (*n*=4). Data represent means±s.d. *t*-test, *P<0.05.

To further determine whether the abnormal osteogenesis in *Med23*_*MSC*_^−/−^ mice results from a primary defect in osteoblast development, we generated an osteoblast-specific *Med23*-deleted mouse model (*Med23*_*ob*_^*−/−*^ mice) by crossing *Med23*^*fl/fl*^ mice with osterix (*Osx*)*-Cre* mice, a line in which *Cre* expression is primarily restricted to osteoblast precursors^21^ (Fig. 2a). Western blot assays showed that MED23 decreased in the bone of both *Med23*_ob_^+/−^ and *Med23*_ob_^−/−^ mice (Supplementary Fig. 3b). Consistent with decline in protein of MED23, histological analysis by alcian blue and alizarin red (ARS) staining showed defects in the skeleton occurring in both *Med23*_ob_^+/−^ and *Med23*_ob_^−/−^ newborns, and more severe in the latter, including abnormality in calvarial and clavicle ossification (Supplementary Fig. 3a). However, *Osx*-Cre transgenic mice have been found to manifest defects in bone phenotype, such as to delay bone mineralization and develop scapula calluses^22^,^23^. To exclude such effect of Osx-Cre, we made a comparison between *Med23*_ob_^−/−^ mice and *Med23*_ob_^+/−^ control littermates that were both in the context of *Osx*-Cre. Again, *Med23*ob^−/−^mice developed runty and underweight compared with age-matched control littermates (Fig. 2b,c). Mice with *Med23* deficiency in osteoblast reproduced the phenotype of *Med23*_*MSC*_^*−/−*^ mice, including impaired membranous ossification of calvarial bones and dysplasia of the clavicles, although the sternum appeared normal. In the *Med23*_*ob*_^*−/−*^ mice, the bone was obviously porous; this feature was more evident in the bones of the skull (Fig. 2d,e). μCT analysis further confirmed the osteopenic phenotype of *Med23*_*ob*_^*−/−*^ mice (Fig. 2f,g). Likewise, relatively lower levels of serum PINP indicated a decreased bone formation rate in *Med23*_*ob*_^*−/−*^ mice (Fig. 2h). Again, *in vivo* and *in vitro* osteoclastogenesis showed comparable levels of osteoclasts between *Med23*_*ob*_^*−/−*^ mice and control littermates (Supplementary Fig. 4a–c). Hence, the *Med23*_*ob*_^*−/−*^ mice recapitulate the defects observed in *Med23*_*MSC*_^*−/−*^ mice with striking fidelity, supporting the conclusion that *Med23* is necessary to the differentiation and function of committed osteoblast precursors.

### *Med23* is required for the osteoblast differentiation of MSCs

**Figure 3:**
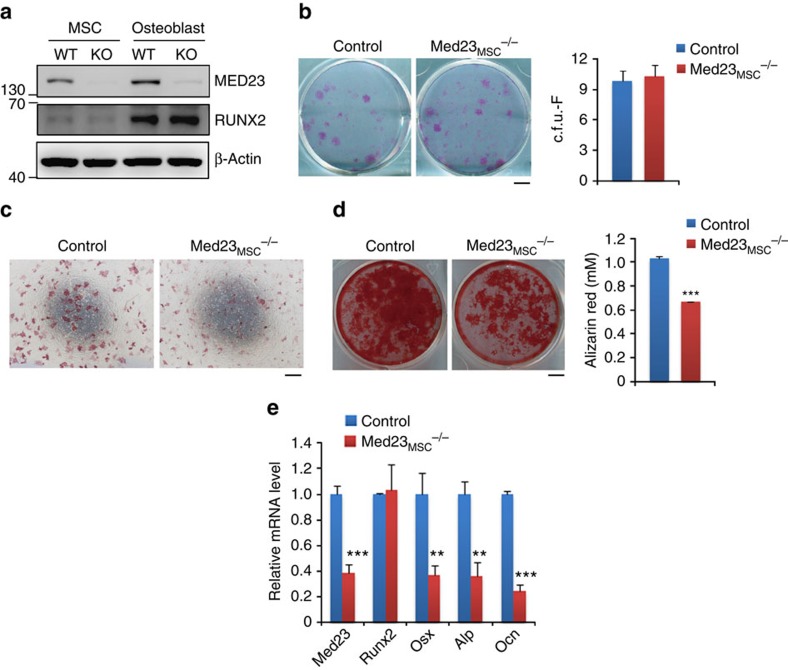
Med23 deficiency inhibits osteoblast differentiation. (**a**) Expression of Med23 and Runx2 in bone marrow-derived mesenchymal stem cells and calvarial osteoblasts from 6 to 8-week-old control and *Med23*_MSC_^−/−^ mice. Isolated cells were expanded and analysed by western blotting. (**b**) c.f.u.-F assay for bone marrow cells from control and *Med23*_MSC_^−/−^ littermates. Representative images of c.f.u.-Fs stained with crystal violet (left, scale bar, 0.5 cm) and quantification of c.f.u.-Fs (right, *n*=3 for each group.). Data represent means±s.d. *t*-test. (**c**) ALP staining of bone marrow mesenchymal stem cells after cultured for 7 days in osteogenic medium. Scale bar, 500 μm. (**d**) c.f.u.-ob assay for bone marrow of control and *Med23*_MSC_^−/−^ littermates. Bone marrow cells were cultured in osteogenic medium for 21 days, followed by staining with Alizarin red (left, scale bar, 0.5 cm) and quantification (right, *n*=3 for each group). Data represent means±s.d. *t*-test.***P<0.001 (**e**) The relative mRNA levels of *Med23*, *Runx2*, *Osx*, *Alp* and *Ocn* were quantified by RT–PCR. Data represent means±s.d. All data represent means±s.d. *t*-test, **P<0.01, ***P<0.001.

Next, we asked whether *Med23* deficiency affects the osteogenic potential of MSCs in a cell-autonomous manner. Primary MSCs were isolated from the bone marrow of control and *Med23*_MSC_^−/−^ littermates, and the deficiency of *Med23* in the MSCs from Med23_MSC_^−/−^ mice was confirmed by western blot assay (Fig. 3a). *Med23* deficiency in MSCs does not appear to alter the cell viability or growth rate, indicated by comparable numbers of colony-forming unit fibroblasts (c.f.u.-F) (Fig. 3b). However, *Med23*^−/−^ MSCs displayed markedly decreased alkaline phosphatase (ALP) activity and mineralization (Fig. 3c,d). In addition, the mRNA levels of osteogenic genes, such as osteocalcin (*Ocn*), were significantly downregulated in *Med23*_MSC_^−/−^ cells (Fig. 3e), while Runx2, a key regulator in bone development, did not change during osteogenesis (Fig. 3a,e). Similarly, impaired osteogenesis was observed in Med23-deficient osteoblasts too (Supplementary Fig. 6). These results indicated that *Med23* functioned necessarily in osteogenesis *in vitro*.

To exclude developmental differences between control and *Med23*^−/−^ primary MSCs, we infected wild-type primary bone marrow MSCs with retroviruses expressing either shRNAs targeting *Med23* or control shRNA. Consistent with the differentiation difference between WT and Med23 KO primary MSCs, shRNA-mediated acute knockdown of *Med23* in MSCs also resulted in decreased osteogenesis, as indicated by ALP and ARS staining (Supplementary Fig. 5a). Real-time PCR analysis showed that *Med23* knockdown reduced the expression of osteogenic marker genes such as *Osx*, *Alp*, collagen type I (*Col1a1*) and *Ocn*. However, noticeably, the expression of Runx2, the master regulator for osteoblast differentiation, was unaffected by *Med23* deficiency (Supplementary Fig. 5b).

Collectively, these results showed that *Med23* is required for osteoblast differentiation. The key osteogenic genes downregulated in *Med23*-deficient cells are known targets of Runx2, suggesting that MED23 might regulate osteoblast differentiation via Runx2.

### *Med23* regulates the gene network of bone development

**Figure 4:**
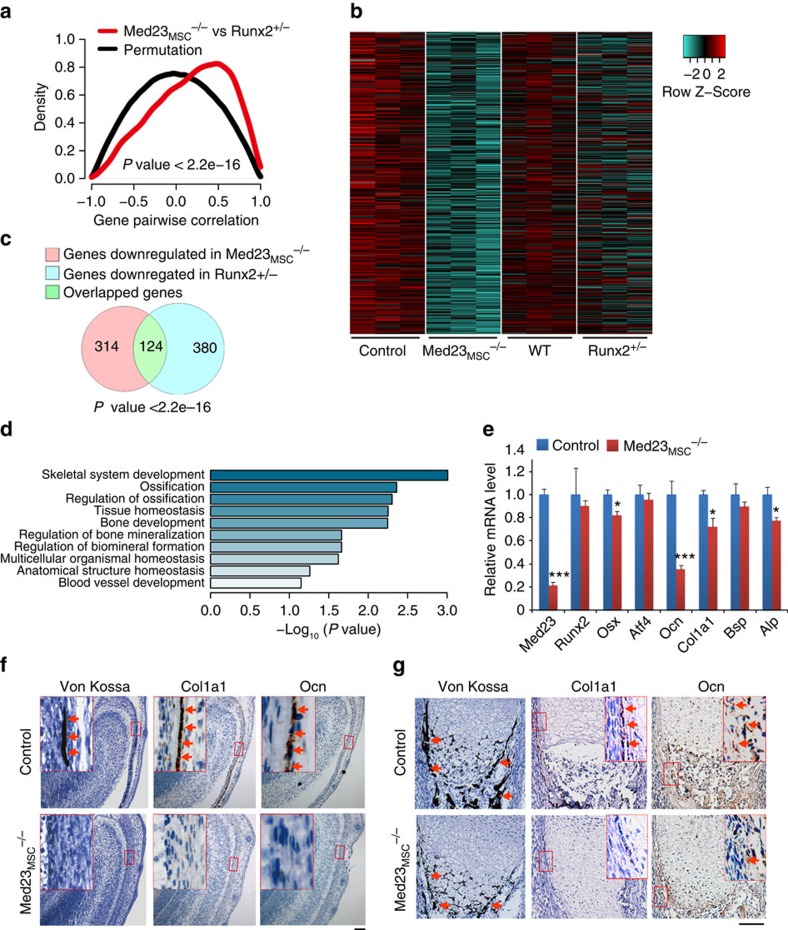
*Med23* regulates expression of genes for osteoblast development. (**a**) Pair-wise correlation analysis of global gene expression in control versus *Med23*_MSC_^−/−^ (*Med23*_MSC_^−/−^ set) and in WT versus *Runx2*^+/−^ (*Runx2*^+/−^ set). Total RNAs were isolated from calvaria bone of control and *Med23*_MSC_^−/−^ newborns and WT and *Runx2*^+/−^ newborns, followed by RNA sequencing analysis. Kolmogorov–Smirnov (K–S) test was used for testing for correlation between *Med23*_MSC_^−/−^ set and *Runx2*^+/−^ set. (**b**) Heat map analysis of downregulated genes in *Med23*_MSC_^−/−^ set and *Runx2*^+/−^ set. (**c**) Analysis of downregulated genes (log_2_ fold change>0.5) common to *Med2*3_MSC_−/− set and *Runx2*^+/−^ set. (**d**) Gene ontology (GO) enrichment analysis of downregulated genes (>1.5-fold) in *Med23*_MSC_^−/−^ set. (**e**) Relative expression level of osteogenic marker mRNAs from parietal bones of P2 control and *Med23*_MSC_^−/−^. Data represent means±s.d. *t*-test, *P<0.05 and ***P<0.001. (**f**) Sections (4 μm) of skull tissues and (**g**) sections (4 μm) of tibia bone from E16.5 control and *Med23*_MSC_^−/−^ embryos were analysed for bone mineralization by von Kossa staining (left), and for expression of *Col1a1* (middle) and *Osteocalcin* (*Ocn*) (right) by *in situ* hybridization. Scale bar, 100 μm.

Despite our observation that the expression of *Runx2*, a master transcription factor for osteoblast differentiation, was not affected by *Med23* deletion during osteogenesis, *Med23*_*MSC*_^*−/−*^ mice appeared to phenocopy the skeletal defects of *Runx2*^+/−^ mice, including delayed closure of the fontanelles, clavicular hypoplasia and decreased bone density^10^, suggesting that *Med23* and *Runx2* share a functional mechanism. To compare the effects of *Med23* and *Runx2* on global gene expression patterns, we performed RNA-seq with total RNA extracted from the calvaria of control and *Med23*_MSC_^*−/−*^ littermates, as well as WT and *Runx2*^+/−^ littermates. Globally, *Med23*_MSC_^−/−^ versus control and *Runx2*^+/−^ versus WT revealed a significantly positive correlation in global gene expression (Fig. 4a). We next found that downregulated genes in *Med23*_MSC_^−/−^ set presented in a similar pattern in *Runx2*+/− set (Fig. 4b). In more details, 438 genes were downregulated >1.5-fold in the *Med23*_MSC_^−/−^ set compared with the corresponding control, and 504 genes were downregulated >1.5-fold in the *Runx2*^+/−^ set compared with the control and a total of 124 genes overlapped between the 2 groups (Fig. 4c). These data further implied that these two molecules may function cooperatively. We then performed a gene ontology (GO) analysis of the set of 1.5-fold significantly downregulated genes from the *Med23*_MSC_^−/−^ sample. The results showed that these genes were enriched for associations with bone development and ossification (Fig. 4d). We verified the expression of osteoblast-specific genes in the calvaria from control and *Med23*_MSC_^−/−^ newborns by real-time PCR and found that osteogenic genes such as *Osx*, *Alp*, *Col1a1* and *Ocn* were significantly reduced in *Med23*_MSC_^−/−^ mice compared with controls, while *Runx2* and *Atf4*, another key factor involved in osteogenesis, were unchanged (Fig. 4e). Consistently, Von Kossa staining and immunohistochemical analyses of bone tissue sections from E16.5 control and *Med23*_MSC_^−/−^ embryos further confirmed the defects in bone formation and impaired osteoblast differentiation *in vivo* (Fig. 4f,g). Similarly, defects in ossification and decrease in osteogenic gene expression were observed in *Med23*_ob_^−/−^ mice (Supplementary Fig. 3c–e). Both the *in vitro* and *in vivo* experiments thus showed that Med23 deletion reduced the expression of multiple *Runx2*-target genes while not affecting the expression of *Runx2* itself. These observations implied that MED23 may participate in osteoblast differentiation and bone development by controlling RUNX2 activity.

### *Med23* modulates Runx2 activity as a cofactor

**Figure 5:**
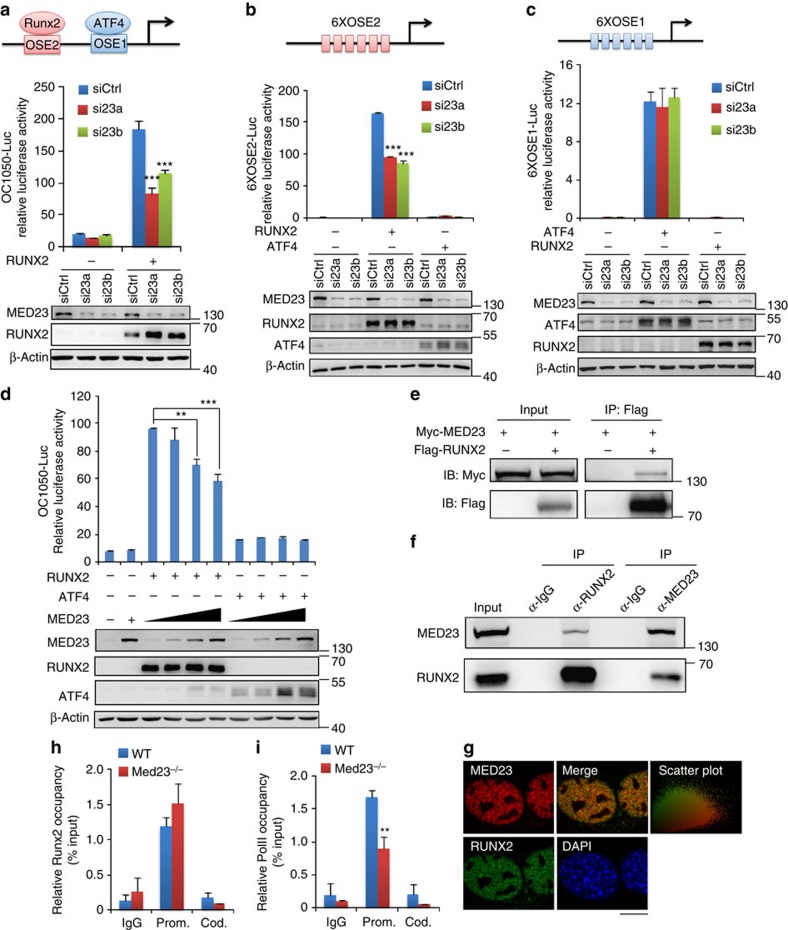
Regulation of RUNX2 transcriptional activity by MED23. (**a**–**c**) Effect of *Med23* deficiency on Runx2-mediated activation of the *osteocalcin* tested with luciferase expression report systems (**a**) OC1050-Luc, (**b**) 6XOSE2-Luc and (**c**) 6XOSE1-Luc. Luciferase assay was performed in control and *Med23* knockdown C310T1/2 cells. *n*=3 for each group, all data represent means±s.d. *t*-test, ***P<0.001 (**d**) Effects of an increasing amount of MED23 on transcriptional activity of Runx2 in C310T1/2 cells with the OC1050-Luc report system. Below is the western blot analysis of MED23 levels in the lysates. *n*=3 for each group, all data represent means±s.d. *t*-test. **P<0.01, ***P<0.001 (**e**) Co-immunoprecipitation (Co-IP) of MED23 with Flag-RUNX2. Flag-RUNX2 expressing plasmid was co-transfected with Myc-Med23 into 293T cells. Whole cell lysate was used for immunoprecipitation and then immunoblotting with indicated antibodies. (**f**) Physical interaction between endogenous RUNX2 and MED23. Co-IP experiment was performed in BMP2-stimulated MC3T3E1 cells. Whole cell lysate was used for immunoprecipitation with anti-RUNX2 or anti-MED23, followed by detection with indicated antibodies by western blot. (**g**) Co-localization assay in primary osteoblast cells. Cells were IF stained with antibodies against MED23 and RUNX2 after stimulated with BMP2 for 24 h. Scale bar, 10 μm. The scatter plot showed FITC and RRX emission intensities were plotted on *x*- and *y*-axes, respectively. Co-localization was analysed by the Volocity software, which showed the coefficient for co-localization of 0.786±0.003 (Pearson's correlation coefficient). (**h**,**i**) ChIP assay for occupation of RUNX2 (**h**) or Pol II (**i**) on the promoter of Osteocalcin. Immortalized WT and *Med23*^−/−^ bone marrow-derived mesenchymal cells were cultured in osteogenic medium for 6 days, followed by fixation and lysation. Chromatin from cell lysates was immunoprecipited with anti-RUNX2 or anti-Pol II and quantified by RT–PCR. ‘Prom' represents the promoter region and ‘Cod' represents the coding region. *n*=3 for each group, all data represent means±s.d. *t*-test, **P<0.01.

We next set out to investigate whether MED23 controls the transcriptional activity of *Runx2*. To this end, we infected murine C3H10T1/2 embryonic fibroblasts with retroviruses expressing control or *Med23* knockdown shRNAs and then transfected the stable cells with a luciferase reporter driven by the *Ocn* promoter (OC1050-Luc), which is a prototypical Runx2-target gene promoter that is widely used as a tool to study the molecular regulation of osteoblast development^24^. In the absence of MED23, RUNX2-dependent activation of the *Ocn* promoter was significantly inhibited (Fig. 5a). The *Ocn* promoter harbours two binding sites: OSE2 and OSE1, which are specifically bound by Runx2 and ATF4, respectively^25^. To discriminate the effects of *Med23* deficiency on the two key factors, we performed the luciferase reporter assay with either 6XOSE2-Luc or 6XOSE1-Luc in control and Med23 shRNA knockdown cells. *Med23* deficiency specifically impaired the Runx2-driven activation of 6XOSE2-Luc but did not affect the ATF4-driven activation of 6XOSE1-Luc, suggesting a specific connection between Med23 and Runx2 (Fig. 5b,c). To further verify the specific regulation of RUNX2 activity by MED23, we performed a titration experiment with low and high doses of MED23 co-transfected with the OC1050-Luc reporter, which showed that RUNX2 activity was repressed gradually with increasing dose of MED23 (Fig. 5d). The repressive effect on RUNX2 activity by overexpression of MED23 was also confirmed by 6XOSE2-Luc reporter system (Supplementary Fig. 7a). By contrast, overexpression of MED23 did not affect ATF4 activity (Fig. 5d and Supplementary Fig. 7b). RUNX2 activity was quenched by high level overexpression of MED23 probably in that the exogenous MED23 may compete with endogenous MED23 within the Mediator complex to prevent the transcription factor *Runx2* from recruiting the Mediator complex.

Cooperative activation of the *Ocn* promoter by MED23 and RUNX2 led us to examine the physical interaction between the two molecules. We transfected 293T cells with tagged Runx2 and Med23 and performed a co-immunoprecipitation (co-IP) assay, which revealed that MED23 physically associated with RUNX2 (Fig. 5e). Consistent with these results, endogenous RUNX2 and MED23 interacted in differentiated MC3T3E1 osteoblastic cells (Fig. 5f). The interaction with MED23 was mediated via the Runt and PST domains of RUNX2 (Supplementary Fig. 8a,b). GST pull-down assay implied that MED23/RUNX2 interaction was likely direct (Supplementary Fig. 8c). Moreover, immunofluorescence staining showed that these two proteins largely colocalized in the nucleus (Fig. 5g). As gene transcription involves a key step in which the activators work with the Mediator complex to recruit RNA Pol II to the gene promoters, we next used a chromatin IP (ChIP) assay to test whether *Med23* deletion affected the recruitment of RNA Pol II by RUNX2 and the binding of RUNX2 to its target's promoter. The result showed that the occupancy of Pol II at the promoter of *Ocn*, a direct target gene of *Runx2*, was markedly decreased in the absence of MED23 while RUNX2 occupancy did not alter, suggesting that *Med23* deficiency impaired the recruitment of Pol II to the promoter of Runx2-target osteogenic marker genes (Fig. 5h). Taken together, these results suggest that the Mediator subunit MED23 acts as a coactivator of Runx2 to regulate osteoblast differentiation.

### *Med23* genetically interacts with *Runx2* during osteogenesis

**Figure 6:**
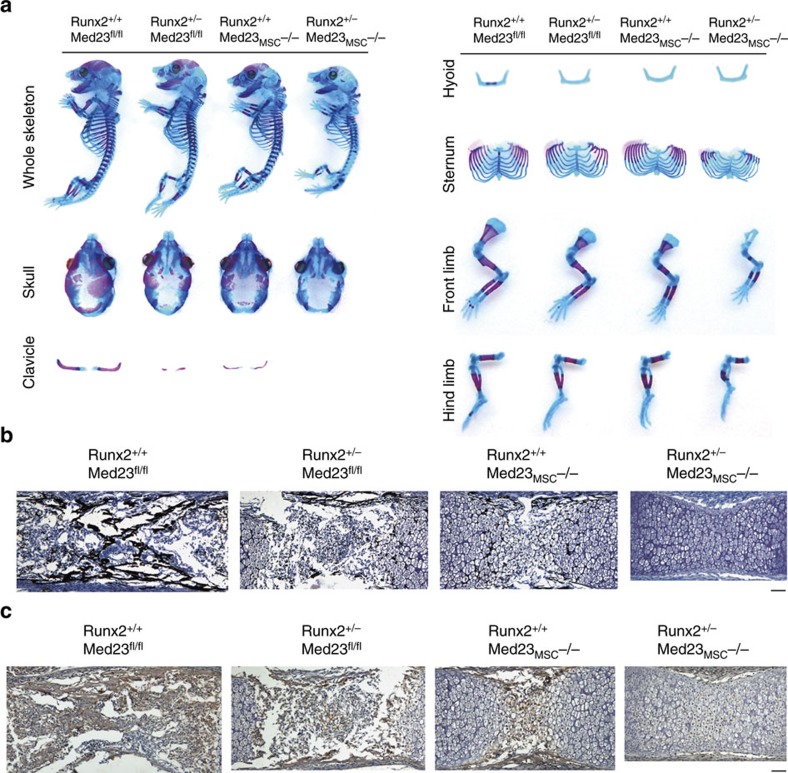
Med23 genetically associates with Runx2. (**a**) Skeletons of E16.5 mice (*Runx2*^+/+^*Med23*^fl/fl^, *Runx2*^+/−^*Med23*^fl/fl^, *Runx2*^+/+^*Med23*_MSC_^−/−^, *Runx2*^+/−^*Med23*_MSC_^−/−^) were double stained by alizarin red/alcian blue. (**b**) Von Kossa staining of humeri from E15.5 mice (*Runx2*^+/+^*Med23*^fl/fl^, *Runx2*^+/−^*Med23*^fl/fl^, *Runx2*^+/+^*Med23*_MSC_^−/−^, *Runx2*^+/−^*Med23*_MSC_^−/−^). Scale bar, 100 μm. (**c**) Immunohistochemistry assay for expression of *Osteocalcin* (*Ocn*) in E16.5 mice (*Runx2*^+/+^*Med23*^fl/fl^, *Runx2*^+/−^*Med23*^fl/fl^, *Runx2*^+/+^*Med23*_MSC_^−/−^, *Runx2*^+/−^*Med23*_MSC_^−/−^). Scale bar, 100 μm.

Based on the *in vitro* findings described above, we next questioned whether MED23 synergized with RUNX2 *in vivo*. We hypothesized that if MED23 acts together with RUNX2 to regulate osteoblast activity, then deletion of *Med23* in parallel with *Runx2* haploinsufficiency *in vivo* should aggravate the skeletal defects observed in *Runx2*^+/−^ mice. To test this hypothesis and since disruption in single copy of *Med23* did not reduce the protein level of MED23, we crossed *Med23*_MSC_^−/−^ mice with *Med23*^fl/fl^*/Runx2*^+/−^ mice and analysed the skeletal phenotypes of E16.5 embryos by alcian blue/ARS double staining. As shown in Fig. 6a, both *Med23*^fl/fl^*/Runx2*^+/−^ mice and *Med23*_MSC_^−/−^ mice showed the previously reported CCD-like skeletal abnormalities observed in heterozygous *Runx2* mutant mice^8^,^10^. *Runx2*^+/−^/*Med23*_MSC_^−/−^ mice displayed significant bone defects, including delayed ossification of the fontanelles and hypoplasia of the clavicles (Fig. 6a). Furthermore, Von Kossa staining revealed reduced ossification of the long bones of E16.5 *Runx2*^+/−^ and *Med23*_MSC_^−/−^ mice compared with control mice. However, *Runx2*^+/−^/*Med23*_MSC_^−/−^ compound mutant mice displayed a more severe reduction in ossification (Fig. 6b). Consistent with the reduced Von Kossa staining, the expression of *Ocn* was decreased significantly in the *Runx2*^+/−^/*Med23*_MSC_^−/−^ compound mutant mice compared with the *Runx2*^+/−^ mice and *Med23*_MSC_^−/−^ mice (Fig. 6c). Taken together, these data indicate a genetic interaction between *Med23* and *Runx2* and provide *in vivo* verification that MED23 can regulate osteoblast function via cooperation with RUNX2.

## Discussion

The present study provides several lines of evidence that *Med23* is required for osteoblast differentiation and bone development. Homozygous loss of *Med23* in mesenchymal progenitors resulted in prominent defects in bone development, including hypoplasia of the clavicles, retarded ossification of the cranial bones and reductions in the trabecular bone and overall bone mass. Furthermore, *Med23* conditional knockout in preosteoblasts largely recapitulated the characteristics of *Med23*_MSC_^−/−^ mice. Phenotypic analysis showed that *Med23* deficiency affected both intramembranous and endochondral bone formation. *In vitro* differentiation experiments indicated that the defective osteogenesis happened in a cell-autonomous manner. In contrast to the impaired osteoblast development, the presence of multinucleated osteoclasts in the mineralized cartilage matrix of *Med23*-deficient mice indicated that osteoclast differentiation occurred normally.

MSCs can differentiate into different lineages, including osteoblasts, adipocytes, chondrocytes and myocytes^1^,^2^. Different transcription factors have been shown to specify distinct cell fates. In addition to the function of *Runx2* in osteoblast differentiation, *PPARγ*, *Sox9* and *MyoD* drive MSCs to differentiate into adipocytes, chondrocytes and myocytes, respectively^7^,^8^,^26^,^27^,^28^. The role of the Mediator complex in *PPARγ*-mediated adipogenesis has been established by observing a direct interaction between MED1 and PPARγ^13^. For *Sox9*, studies from the zebra fish model indicated that Trap230/MED12 can function as a coactivator for SOX9 during cartilage development, with *Trap230*/*Med12* mutant zebra fish strikingly resembling the Sox9 mutant phenotype^29^. Our previous study demonstrated that MED23 represses smooth muscle cell differentiation while facilitating adipocyte differentiation in multipotent MSCs^16^, indicating that a single Mediator complex subunit is able to regulate the differentiation of MSCs into various cell lineages, either positively or negatively, by cooperating with lineage-specifying transcription factors such as ELK1 and MAL.

To date, there have been no reports of a direct role for the Mediator complex in RUNX2 function. Our study demonstrated that MED23 can act together with RUNX2 to drive osteoblast differentiation. To the best of our knowledge, this is the first report of a direct role for the Mediator complex in modulating RUNX2 function. We provide evidence that the Mediator complex subunit MED23 partially regulates the transcriptional activity of RUNX2 and is required for osteoblast differentiation and bone development. First, homozygous loss of *Med23* in mesenchymal progenitors resembled the bone phenotype of *Runx2* heterozygous mice, resulting in prominent defects in bone development, with hypoplasia of the clavicles, retarded ossification of the cranial bones and reduction in the trabecular bone and overall bone mass. Moreover, *Med23* conditional knockout in preosteoblasts largely recapitulated the characteristics of *Med23*_MSC_^−/−^ mice, with abnormalities in multiple skeletal elements. Interestingly, *Med23* deficiency did not alter RUNX2 expression but instead caused the downregulation of multiple *Runx2*-regulated genes, such as *Osx* and *Ocn*. Reporter assays revealed that *Med23* deficiency impaired Runx2 transcriptional activity but did not affect the activity of ATF4, another key regulator of osteoblast differentiation. Biochemical analyses further established the physical association between RUNX2 and MED23. Finally, *Med23* deletion further aggravated the defective skeletal phenotype of *Runx2*^+/−^ mice. In addition to the function in osteoblasts, *Runx2* is also found to control the maturation of chondrocytes through *Runx2* conditional knockout in chondrocytes by *Col2a1-Cre*^30^,^31^. Histological analysis of tibia showed that chondrocytes were arrested during terminal maturation in *Med23*_MSC_^−/−^ mice compared with control littermates as evidenced by less endochondral ossification and smaller hypertrophic chondrocytes in *Med23*_MSC_^−/−^ mice (Supplementary Fig. 10a–c). All the results described above provide *in vivo* genetic evidence that MED23 is a cofactor of RUNX2.

*Runx2* mutations are known to underlie human CCD, an autosomal-dominant heritable skeletal disease that is typically characterized by open or delayed closure of calvarial fontanelles and clavicle hypoplasia^9^,^32^,^33^. This phenotype can be reproduced in heterozygous *Runx2* mutant mice^10^. More precisely, a 70% reduction in *Runx2* levels also generates the CCD phenotype in mice^34^. Indeed, human bone marrow-derived MSCs with Med23 deficiency were retarded to differentiate into osteoblasts, which might imply that MED23 plays a role in human skeletal development and its mutation may relate to human bone diseases (Supplementary Fig. 9). In addition, the dysregulation of Runx2 activity through manipulation of numerous nuclear factors, for example, TAZ, MAF and Satb2, causes abnormal osteoblast differentiation and bone development^11^,^35^,^36^. These studies indicate that the *Runx2*-dependent transcriptional output is under fine surveillance via the cooperation of many factors. Our study demonstrated that the Mediator subunit MED23 acts as a cofactor of RUNX2 in the regulation of osteoblast differentiation and bone development, providing an insight into the regulation of RUNX2 activity and skeletal dysplasias such as CCD.

## Methods

### Mice

*Med23*-floxed mice were generated by homologous recombination^16^,^17^. Briefly, the exons 5–7 were flanked by two loxP sites. After targeting vector delivery, ES cells were screened by PCR. *Med23*-floxed mice were backcrossed with C57/BL6J mice for at least six generations. *Prx1-Cre* and *Osx-Cre-ER* mice were purchased from the Jackson Laboratory. *Runx2*^*+/*−^ mice were kindly provided by Professor Laurie Glimcher's lab. All mice were maintained under specific pathogen-free conditions. All animal experimental procedures were approved by the Institutional Animal Care and Research Advisory Committee of the Shanghai Institute of Biochemistry and Cell Biology.

### Analysis of bone phenotypes

Skeletal preparations were double stained with alcian blue and ARS^8^,^10^. Briefly, embryos or newborns were eviscerated and the skin was removed. After fixation with 95% ethanol for 3 days, embryos or newborns were stained for 3 days in Alcian blue solution. Then they were fixed and cleared with 95% ethanol for three times and each 1.5 h, followed by treatment of 2% KOH for 3–4 h. After stained with ARS solution for 3–4 h, skeletons were cleared in 1%KOH/20% glycerol. For histological analysis, bone tissues were fixed in 4% paraformaldehyde (PFA) and then embedded in paraffin. For embryonic mice, 4 μm tissue sections were used for Von Kossa staining, DIG labelled *in situ* hybridization (Roche) and immunohistochemical staining (Dako). For postnatal mice, bone tissues were fixed in 4% PFA and decalcified for 2 weeks prior to paraffin embedding. Tissue sections (4 μm) were used for TRAP staining according to the standard protocol.

### Measurement of PINP concentrations

We determined serum concentrations of PINP using the mouse PINP EIA kit (Immunodiagnostic Systems) according to the instructions provided.

### Micro CT analysis

Mouse hind limbs were harvested, soft tissues were removed and the remaining tissues were stored in 70% ethanol. Scanning was performed with a Skyscan1076 instrument, and 36 slides (18 μm each) immediately below the growth plate in the distal metaphysis of the femur were used for quantification of the bone parameters.

### Cell culture

C310T1/2 (ATCC) and 293T (ATCC) cells were maintained in DMEM containing 10% FBS. MC3T3E1 (ATCC) cells were maintained in Minimum Essential Medium α (α-MEM) containing 10% FBS. MSCs were isolated from bone marrow samples of 6- to 8-week-old mice and cultured in α-MEM containing 15% FBS. All cells were cultured in a 5% CO_2_ humidified incubator at 37 °C. For the c.f.u.-F assay, 2 × 10^6^ bone marrow-derived MSCs or 1 × 10^2^ osteoblast cells from calvaria were plated on each well of a six-well plate, cultured for 2 weeks and then fixed in 4% PFA and stained with 0.5% crystal violet, followed by counting the stained colonies. For *in vitro* osteoblast differentiation, MSCs were cultured in osteogenic medium (10% FBS with 50 μg ml^−1^ ascorbic acid, 10 nM dexamethasone and 10 mM β-glycerophosphate) and subjected to ALP staining on day 7 and ARS staining on day 21. Human bone marrow-derived MSCs were cultured and induced to differentiate into osteoblasts according to the protocol from ScienCell.

### Transient transfection and luciferase reporter assay

C3H10T1/2 cells were seeded overnight at 4 × 10^4^ cells per well into a 24-well plate and transfected by Lipofectamine 2000 (Life Technologies) with a luciferase reporter plasmid and pRL-TK (Promega) along with various expression constructs, as indicated. All wells were supplemented with control empty expression vector plasmids to keep the total amount of DNA constant. At 36–48 h post transfection, the cells were harvested and subjected to dual-luciferase reporter assays according to the manufacturer's protocol (Promega).

### IP and immunoblotting

293T cells were seeded at 6 × 10^6^ cells per 10 cm dish and cultured overnight. At 36–48 h after transfection with Lipofectamine 2000, cells were harvested and lysed in lysis buffer (1% NP-40, 10% glycerol, 135 mM NaCl, 20 mM Tris, pH 8.0) supplemented with protease inhibitors. Lysates were subjected to IP with anti-Flag antibodies (M2, Sigma) at 4 °C overnight, followed by washing in lysis buffer, SDS–PAGE electrophoresis and immunoblotting with the indicated antibody (1:2,000, *anti-Myc*, Sigma). To investigate endogenous protein–protein interactions, MC3T3E1 cells were cultured for 4 days in α-MEM with 10% FBS supplemented with 100 ng ml^−1^ BMP2, 50 μg ml^−1^ ascorbic acid and 10 mM β-glycerophosphate. Two hours prior to harvest, MG132 (10 μM, Sigma) was added to all cultures. Harvested cells were subjected to lysis and IP with anti-RUNX2 (Sigma), anti-MED23 (Novus) or control IgG, followed by washing in lysis buffer, SDS–PAGE electrophoresis and immunoblotting with the indicated antibody (Anti-MED23, 1:1,000, BD Biosciences; anti-Runx2, 1:1,000, MBL). Primary bone marrow MSCs or bone tissues were lysed in RIPA buffer (50 mM Tris pH7.4, 150 mM NaCl, 1% Triton X-100, 1% sodium deoxycholate, 0.1% SDS) in presence of protease inhibitors. Whole cell lysate were centrifuged and then subjected to western blots according to standard protocols. Uncropped results for all western blots are shown in Supplementary Fig. 11.

### *In vitro* interaction assay

GST or GST-RUNX2 recombined proteins were expressed in *Escherichia coli* BL21, followed by purification according to the instructions of the manufacturer (GE). His-Flag-MED23 was expressed using the Bac-to-Bac baculovirus expression system and purified by Ni-NTA agarose beads (Invitrogen). Purified GST or GST-Runx2 was incubated with purified MED23 and subjected to GST pull-down experiment . The bound proteins were analysed by SDS–10% PAGE and immunoblotting with indicated antibodies (Anti-GST, 1:1,000, Santa Cruz; anti-MED23, 1:1,000).

### Immunofluorescence staining and co-localization analysis

Primary osteoblast cells were plated on cover slips for 24 h in α-MEM with 10% FBS with BMP2 (100 ng ml^−1^) prior to staining. After removal of culture media, cells were washed with PBS, fixed in 4% PFA, permeabilized with 0.2% Triton X-100 for 10 min and then blocked with 2% BSA in PBS for 1 h. Anti-MED23 (1:100) and anti-RUNX2 (1:200) primary antibodies were diluted in the blocking solution and applied overnight at 4°C. After PBS wash for three times, secondary antibodies (Jackson Laboratory) were diluted (1:100) in blocking buffer and applied for 1 h at room temperature. After PBS wash for three times, nuclei were stained with DAPI (1:4,000) for 4 min. Co-localization analysis were performed using Volocity Software (PerkinElmer).

### Real-time PCR analysis

Total RNA was isolated from bone tissue or cells with TRIzol reagent (Life Technologies). Complementary DNA was generated using M-MLV reverse transcription kit (Promega). Real-time PCR was conducted in triplicate with SYBR Premix Ex Taq (Takara). The level of the endogenous mRNA was normalized to the level of *Gapdh* mRNA using the 2^−ΔCT^ method. Specific primer sequences are listed in the Supplementary Table 1.

### ChIP assay

Cells were cross-linked with 1% formaldehyde in culture media for 10 min at room temperature, followed by adding 0.125 M glycine to quench the cross-linking. Cells were washed and scraped in cold PBS and harvested in ChIP lysis buffer (50 mM Tris-HCl, pH 7.4, 1% SDS, 10 mM EDTA). Cell lysates were then sonicated to shear chromatin. After that, cell lysates were centrifuged and 100 μl supernatant was diluted with 900 μl dilution buffer (16.7 mM Tris-HCl, pH 8.0, 0.01% SDS, 1.1% Triton X-100, 1.2 mM EDTA, 167 mM NaCl) and then incubated with 2 μg anti-RUNX2 or 2 μg anti-Pol II (Santa Cruz) or non-immune Rabbit IgG overnight at 4° C. About 20 μl Protein G magnetic beads (Life technologies) were added for another 4 h on a rotating wheel. Chromatin then was immunoprecipitated, decrosslinked at 65 °C and treated with Proteinase K. Precipitated DNA was extracted and quantified by real-time PCR. All values were normalized to input. Specific primer sequences were listed in Supplementary Table 1.

### Retrovirus infection

Retroviruses expressing RNA interference (RNAi) oligonucleotides were generated by transfection of recombinant pSiren-RetroQ and pCL10A1 helper plasmids into 293T cells using Lipofectamine 2000. Culture media were changed 24 h after transfection and the supernatants containing retroviruses were harvested 48 h later and passed through a 0.45-μm filter. Filtered supernatants were supplemented with 6 μg ml^−1^ polybrene and added to the cells grown in six-well plates for centrifugation at 2,500 r.p.m. for 1.5 h at 30 °C. About 24 h after spin infection, infected cells were then selected in the presence of puromycin: primary MSCs at 5 μg ml^−1^ and C3H10T1/2 at 8 μg ml^−1^. The RNAi oligo sequences are listed in Supplementary Table 2.

### RNA-Seq and GO analysis

Total RNA was isolated with TRIzol from the parietal bone of P2 control mice (*n*=3) and *Med23*_MSC_^−/−^ littermates (*n*=3) and from the parietal bone of P2 WT mice (*n*=3) and Runx2^+/−^ mice (*n*=3). Complementary DNA library preparation and sequencing were performed according to the Illumina standard protocol. GO analysis was performed with the DAVID online tool. Top GO categories were selected according to the *P* values. RNA-seq data in this study have been deposited at Gene Expression Omnibus (GEO) (http://www.ncbi.nlm.nih.gov/gen/) under accession ID GSE 77007.

### Statistical analysis

Statistical analyses were performed with two-tailed, unpaired Student's *t*-test. Kolmogorov–Smirnov test was used to test for comparing cumulative distributions of two data sets (Fig. 4a).

## Additional information

**Accession codes:** RNA-seq data has been deposited in the Gene Expression Omnibus (GEO) Data Bank (http://www.ncbi.nlm.nih.gov/gen/) under accession code ID GSE77007.

**How to cite this article:** Liu, Z. *et al*. Mediator MED23 cooperates with RUNX2 to drive osteoblast differentiation and bone development. *Nat. Commun.* 7:11149 doi: 10.1038/ncomms11149 (2016).

## Supplementary Material

Supplementary Information Supplementary Figures 1-11 and Supplementary Tables 1-2
