# Supplementary material for: Mediator MED23 cooperates with RUNX2 to drive osteoblast differentiation and bone development
Source: Nat Commun. 2016 Apr 1;7:11149. doi: 10.1038/ncomms11149 (PMC4821994; doi:10.1038/ncomms11149)
Supplement: Supplementary Information — Supplementary Figures 1-11 and Supplementary Tables 1-2 [file ncomms11149-s1.pdf]

Supplementary Figure 1.

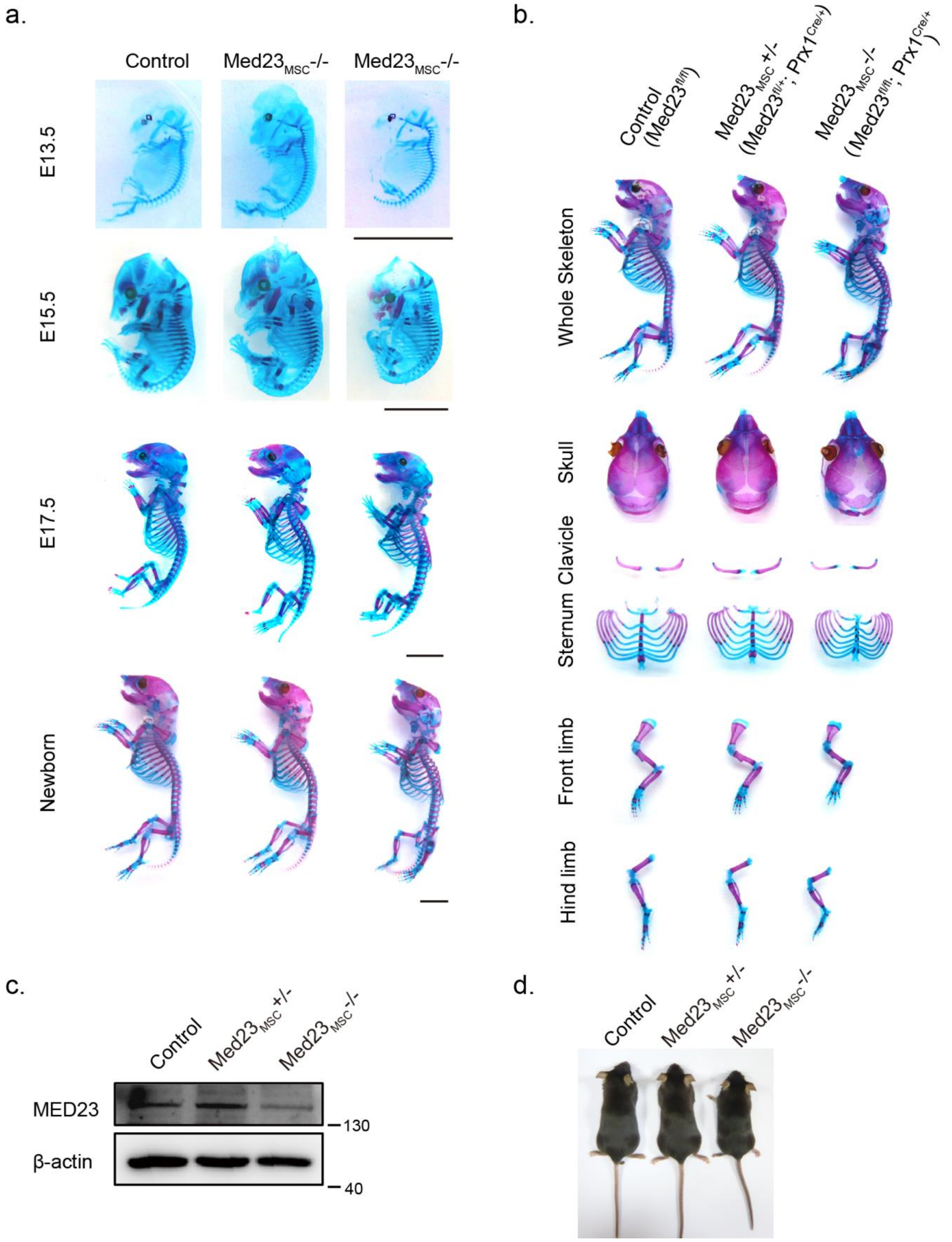

Supplementary Figure 1. Examination of skeletons from control, *Med23*$_{MSC}^{+/-}$ and *Med23*$_{MSC}$-/- mice.

(**a**) Alcian blue and alizarin red staining of whole skeleton from E13.5 embryos to newborns of control (*Med23*$^{fl/fl}$), *Med23*$_{MSC}^{+/-}$ and *Med23*$_{MSC}^{-/-}$ mice. Scale bar=0.5cm. (**b**) Gross appearance of control, *Med23*$_{MSC}^{+/-}$ and *Med23*$_{MSC}^{-/-}$ mice at P0. *Med23*$_{MSC}^{+/-}$ mice appears as normal as control littermate while *Med23*$_{MSC}^{-/-}$ mice has a small stature with short legs. (**c**) Western blot analysis for MED23 level in long bone from control, *Med23*$_{MSC}^{+/-}$ and *Med23*$_{MSC}^{-/-}$ mice at 3 weeks. (d) Gross appearance of one-month control, *Med23*$_{MSC}^{+/-}$ and *Med23*$_{MSC}^{-/-}$ mice.

Supplementary Figure 2.

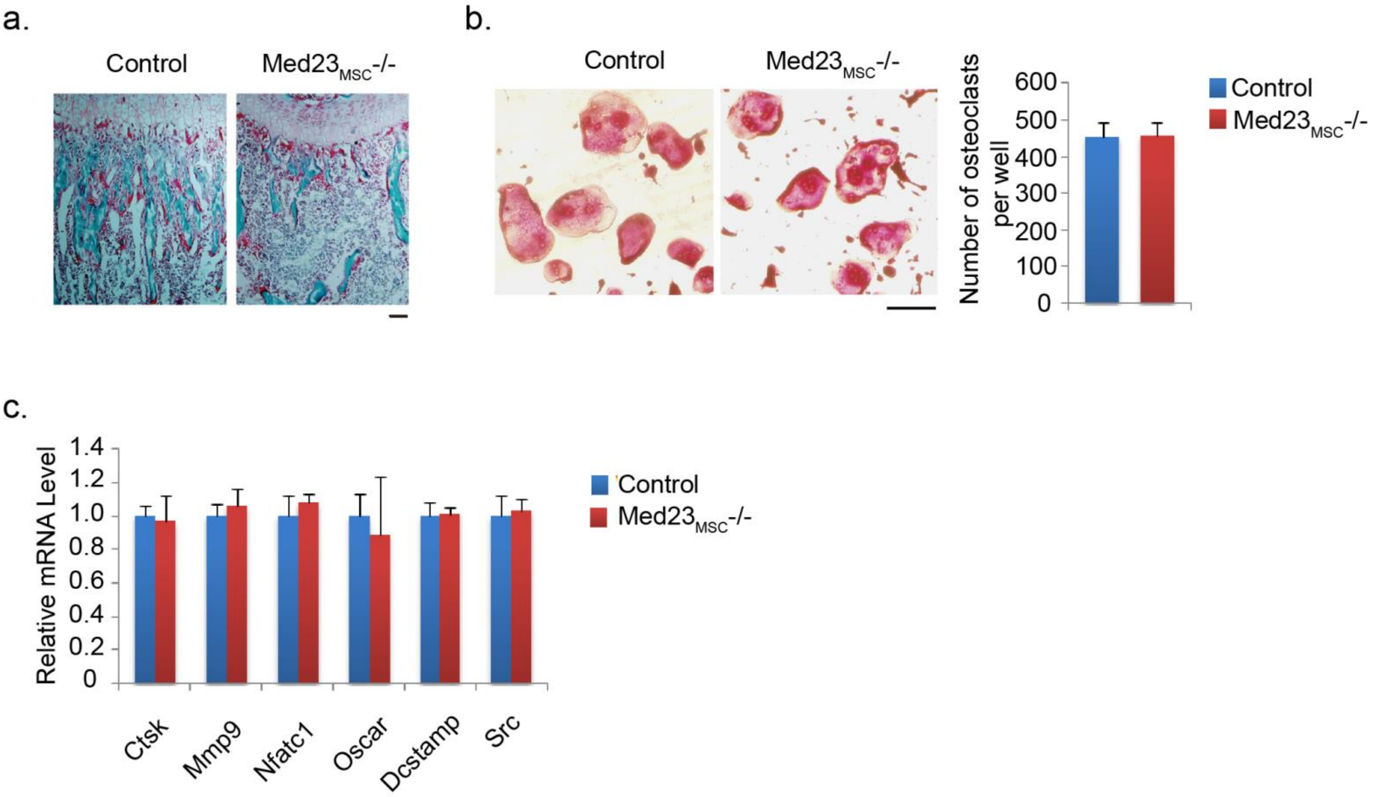

Supplementary Figure 2. In vivo and in vitro osteoclastogenesis in control and *Med23*$_{MSC}^{-/-}$ mice (**a**) Trap staining of trabecular bone of 1-month-old control (*Med23*$^{fl/fl}$) mice and *Med23*$_{MSC}^{-/-}$ littermate. Scale bar= 50 μm. (**b**) In vitro differentiation of osteoclasts from bone marrow cells from control and Med23$_{MSC}^{-/-}$ mice. Data represent mean ± s.d. (n=3 for each group, *t*-test). Scale bar= 200 μm. (**c**) Expression of osteoclastic genes analyzed by real time PCR. Data represent means ± s.d. (n=3 for each group, *t*-test).

Supplementary Figure 3.

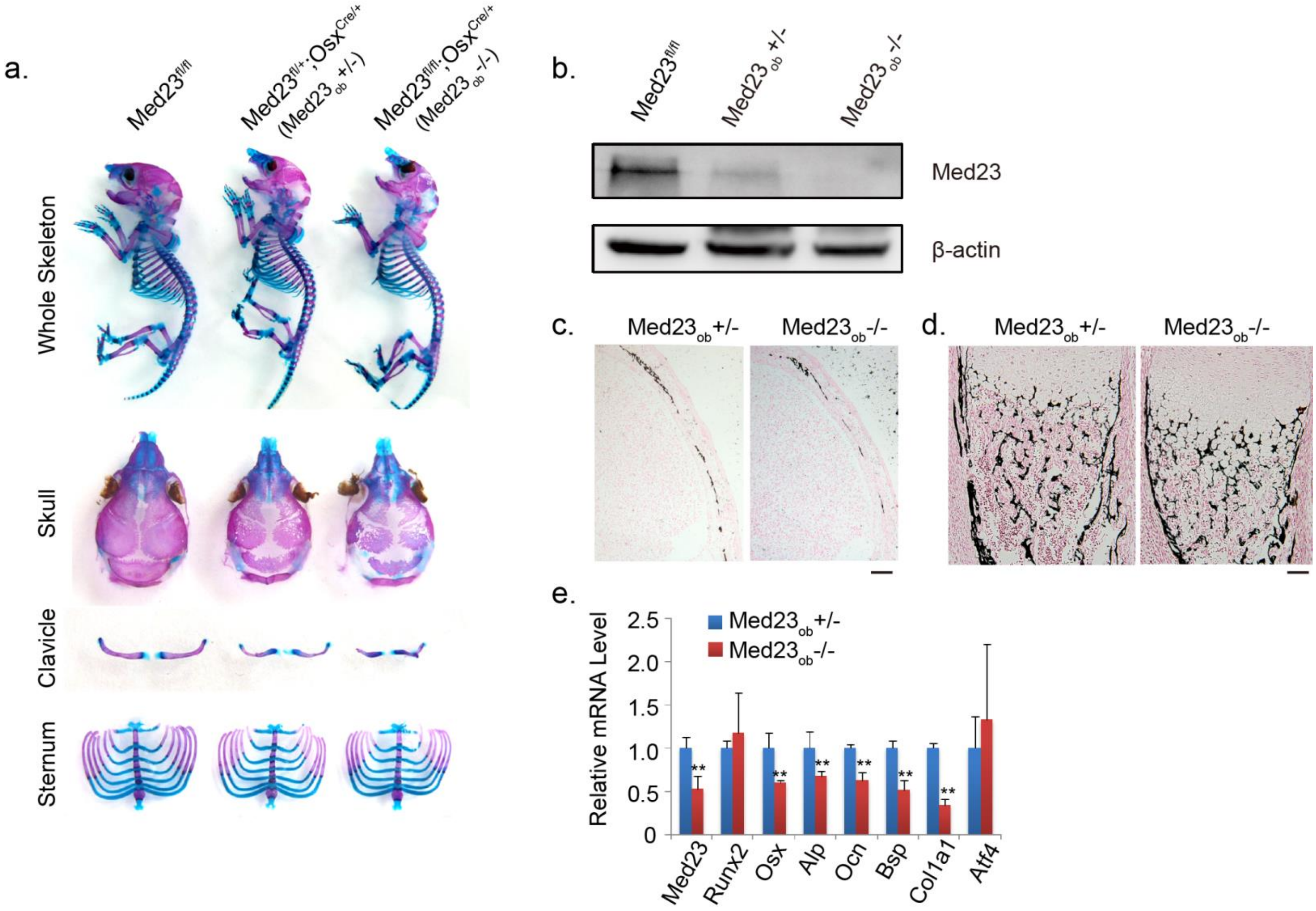

Supplementary Figure 3. Molecular and histological analysis of bone from control and *Med23*$_{ob}$-/- mice. (**a**) Examination of skeleton from *Med23*$_{ob}^{+/-}$ and *Med23*$_{ob}^{-/-}$ mice newborns by alcian blue and alizarin red staining. (**b**) Western blot analysis for MED23 level in long bone from *Med23*$^{fl/fl}$, *Med23*$_{ob}^{+/-}$ and *Med23*$_{ob}^{-/-}$ mice at 3 weeks. (**c-d**) Von Kossa staining of parietal bone (c) and tibia (d) from E16.5 *Med23*$_{ob}^{+/-}$ and *Med23*$_{ob}^{-/-}$ embryos. Scale bar=100μm. (**e**) Real time PCR analysis of expression of osteoblastic genes in calvarial bone. Data represent means ± s.d. (n=3 for each group, *t*-test).

Supplementary Figure 4.

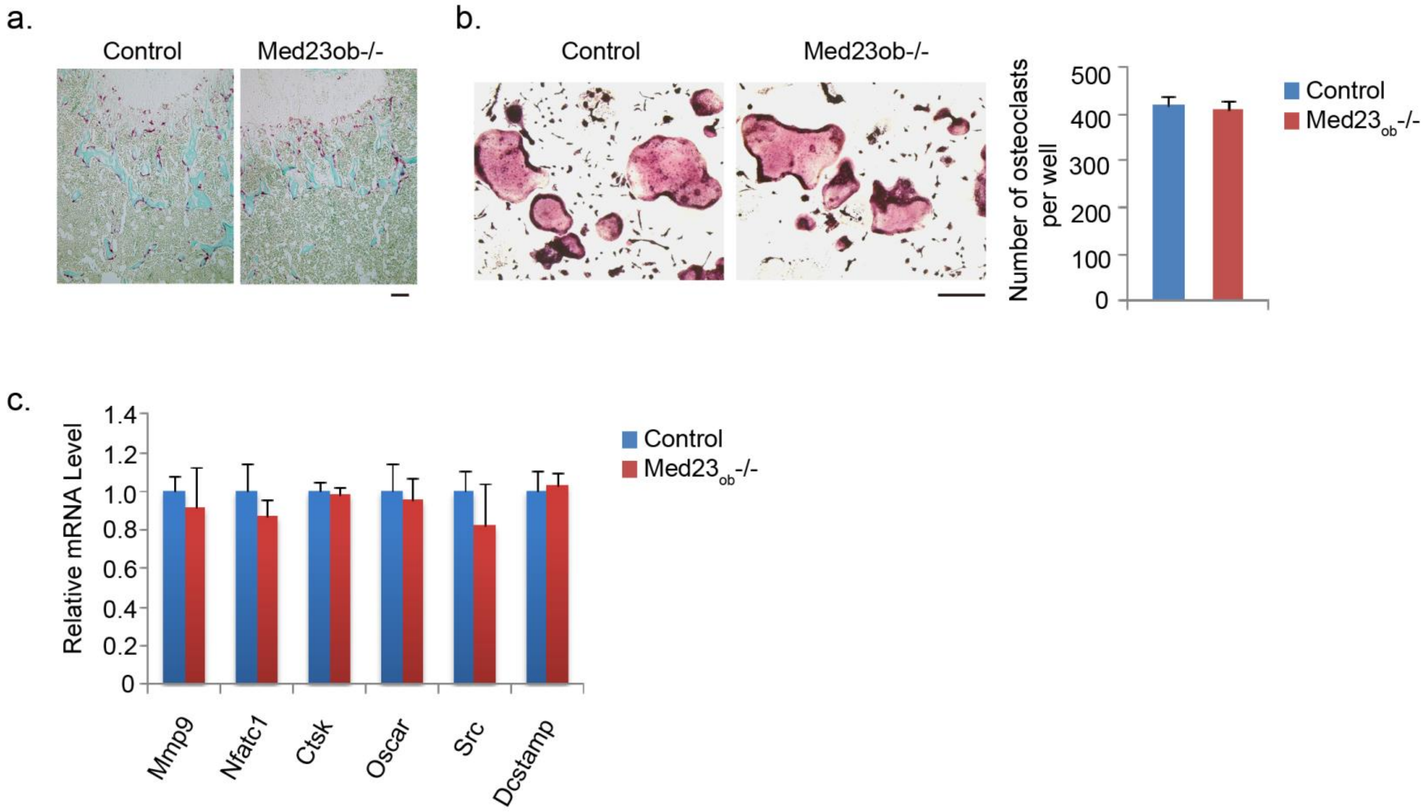

Supplementary Figure 4. In vivo and in vitro osteoclastogenesis in control (*Med23*$_{ob}^{+/-}$) and *Med23*$_{ob}^{-/-}$ mice

(**a**) Trap staining of trabecular bone of 1-month-old control mice and *Med23*$_{ob}^{-/-}$ littermate. Scale bar=50μm. (**b**) In vitro differentiation of osteoclasts from bone marrow cells from control and Med23$_{ob}^{-/-}$ mice. Data represent means ± s.d. (n=3 for each group, *t*-test). Scale bar= 200μm. (**c**) Expression of osteoclastic genes analyzed by real time PCR. Data represent means ± s.d. (n=3 for each group, *t*-test).

Supplementary Figure 5.

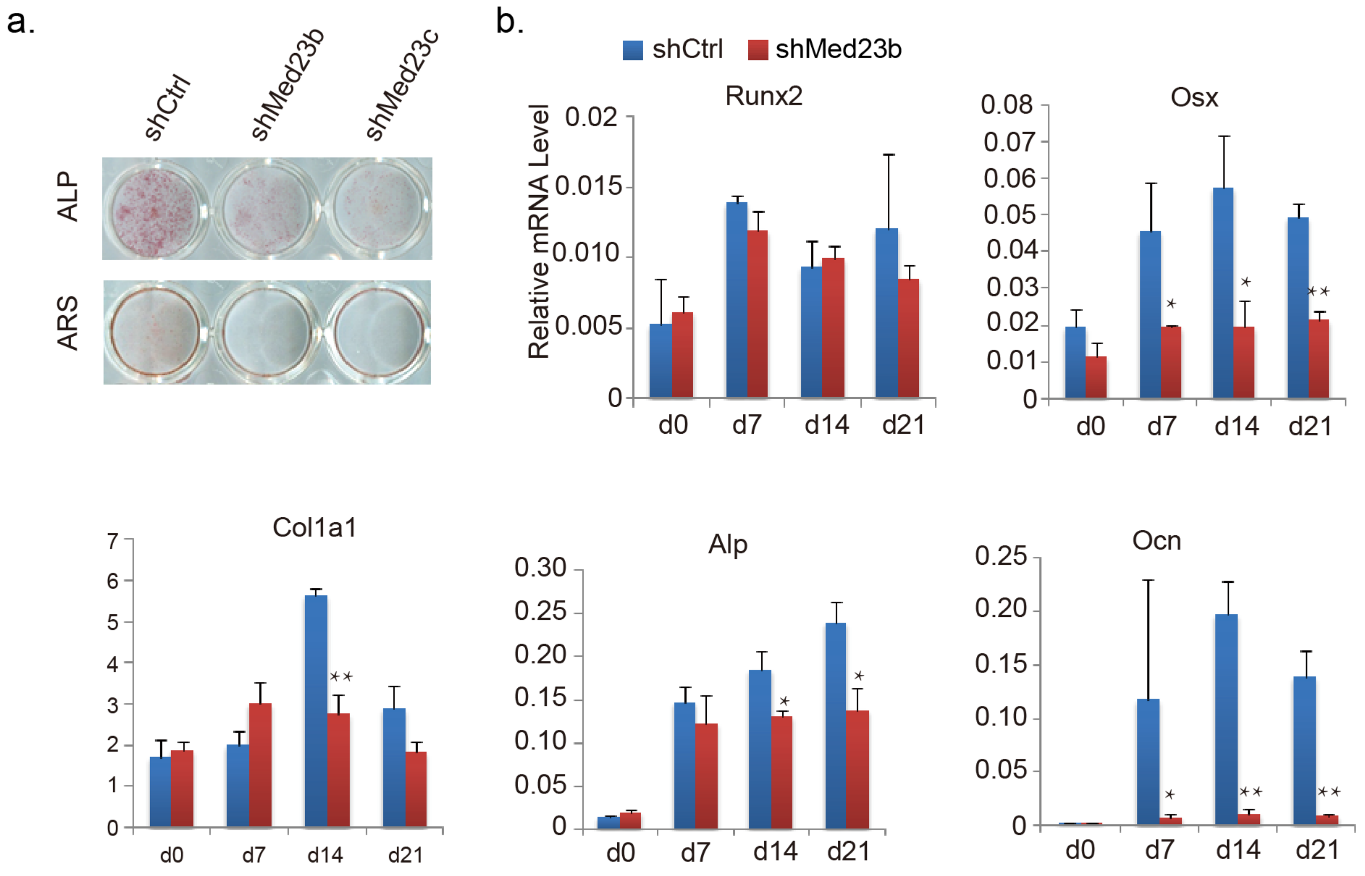

Supplementary Figure 5. The effect of Med23 deficiency on osteogenesis of bone marrow-derived mesenchymal stem cells in vitro

(**a**) Alkaline phosphatase (ALP) staining and alizarin red (ARS) staining. Bone marrow-derived mesenchymal stem cells were isolated and infected with retrovirus expressing control or *Med23* shRNA. After screened by puromycin, infected cells were cultured in osteogenic medium for up to 21 days. Stainings were performed at day 7 and day 21 respectively. (**b**) RT-PCR analysis of mRNA levels of osteogenic genes (*Runx2*, *Osx*, *Alp* and *Ocn*) at indicated time points. All data represent means± s.d (n=3 for each group, *t*-test). *P<0.05, **P<0.01.

Supplementary Figure 6.

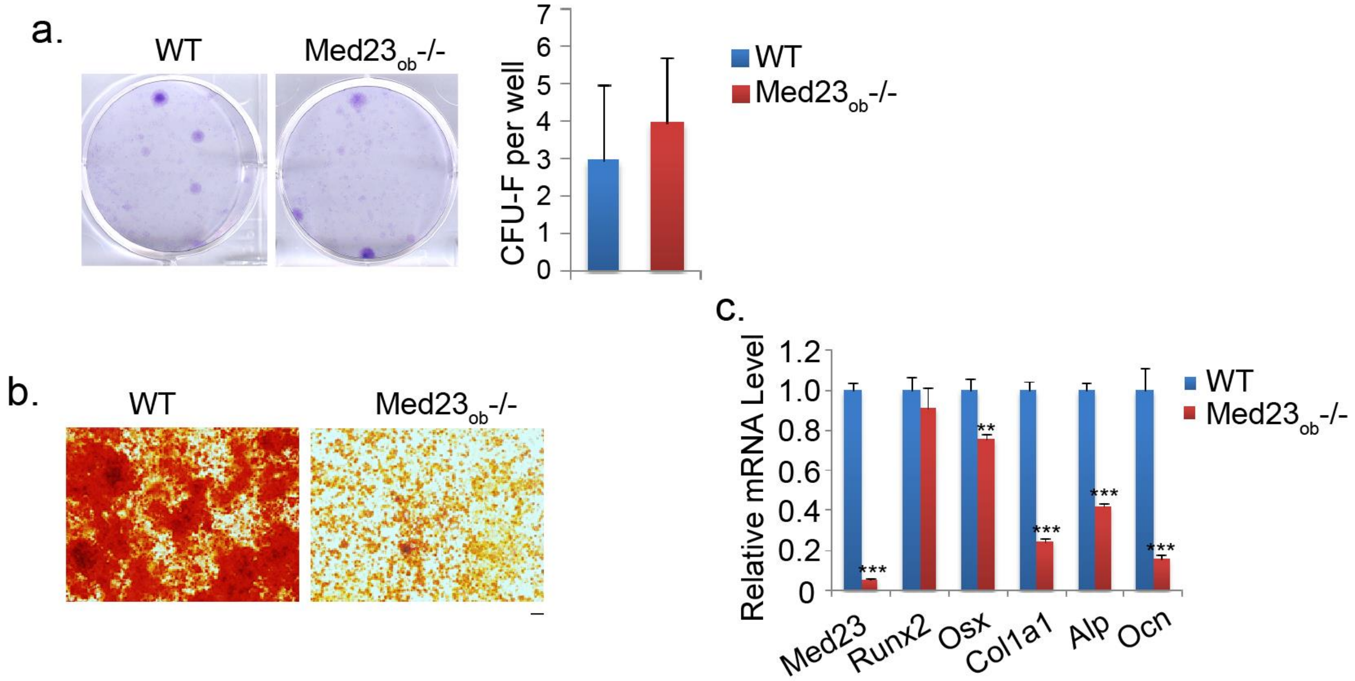

Supplementary Figure 6. In vitro osteogenesis of primary osteoblasts from wild type and *Med23*$_{ob}^{-/-}$ mice (**a**) CFU-F assay for 6-well plate cultured bone marrow cells from wild type and *Med23*$_{ob}^{-/-}$ newborns. Data represent means ± s.d. (n=3 for each group, *t*-test). (**b**) Mineralization by analysis of alizarin red staining. Scale bar= 100µm. (**c**) Expression of osteoblastic genes analyzed by real time PCR. Data represent means ± s.d. (n=3 for each group, *t*-test). ** P<0.01, *** P<0.001.

Supplementary Figure 7.

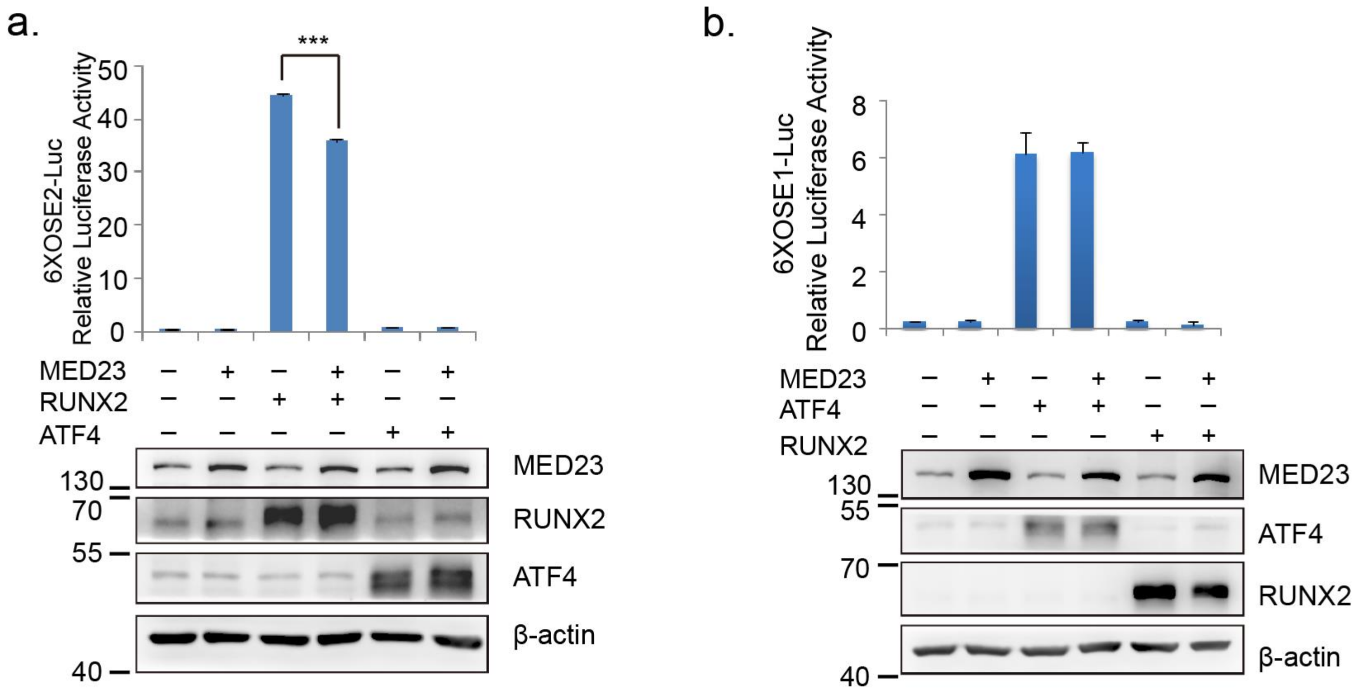

Supplementary Figure 7. Effect of MED23 overexpression on RUNX2 or ATF4-mediated luciferase activity

(**a**) For effect on transcriptional activity of RUNX2, the luciferase assay was performed in control and *Med23* knockdown C310T1/2 cells with the 6XOSE2-Luc report system. Data represent means ± s.d. (n=3, *t*-test). *** *P*<0.001. (**b**) For effect on transcriptional activity of ATF4, the luciferase assay was performed in control and *Med23* knockdown C310T1/2 cells with the 6XOSE1-Luc report system. Data represent means ± s.d. (n=3, *t*-test).

Supplementary Figure 8.

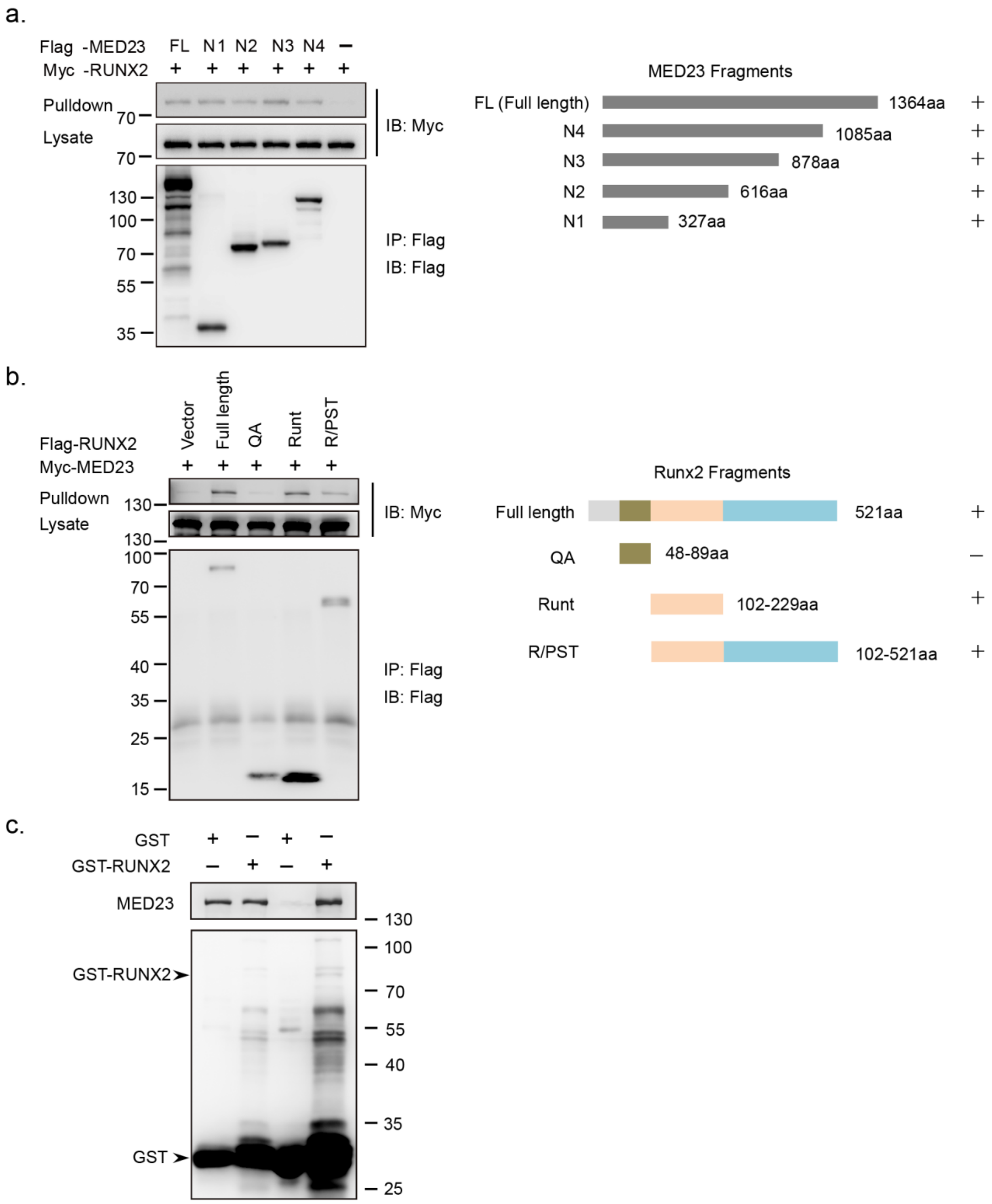

Supplementary Figure 8. Physical interaction of MED23 and RUNX2

(**a**) 293T cells were transfected with expression of encoding Flag-tagged MED23 deletion mutants and Myc-RUNX2. Flag-MED23 was immunoprecipitated with the anti-Flag antibody, followed by western blotting to detect Myc-RUNX2. (**b**) 293T cells were transfected with expression of encoding Flag-tagged RUNX2 deletion mutants and Myc-MED23. Deletion mutants of Flag-RUNX2 were coimmunoprecipitated with Myc-MED23 using the anti-Flag antibody. (**c**) Baculovirus expressed His-Flag-MED23 was purified by Ni-NTA beads, followed by incubation glutathione S-transferase (GST) or GST-RUNX2. The bound proteins were precipitated with glutathione agrose, followed by western blotting with indicated antibodies.

Supplementary Figure 9.

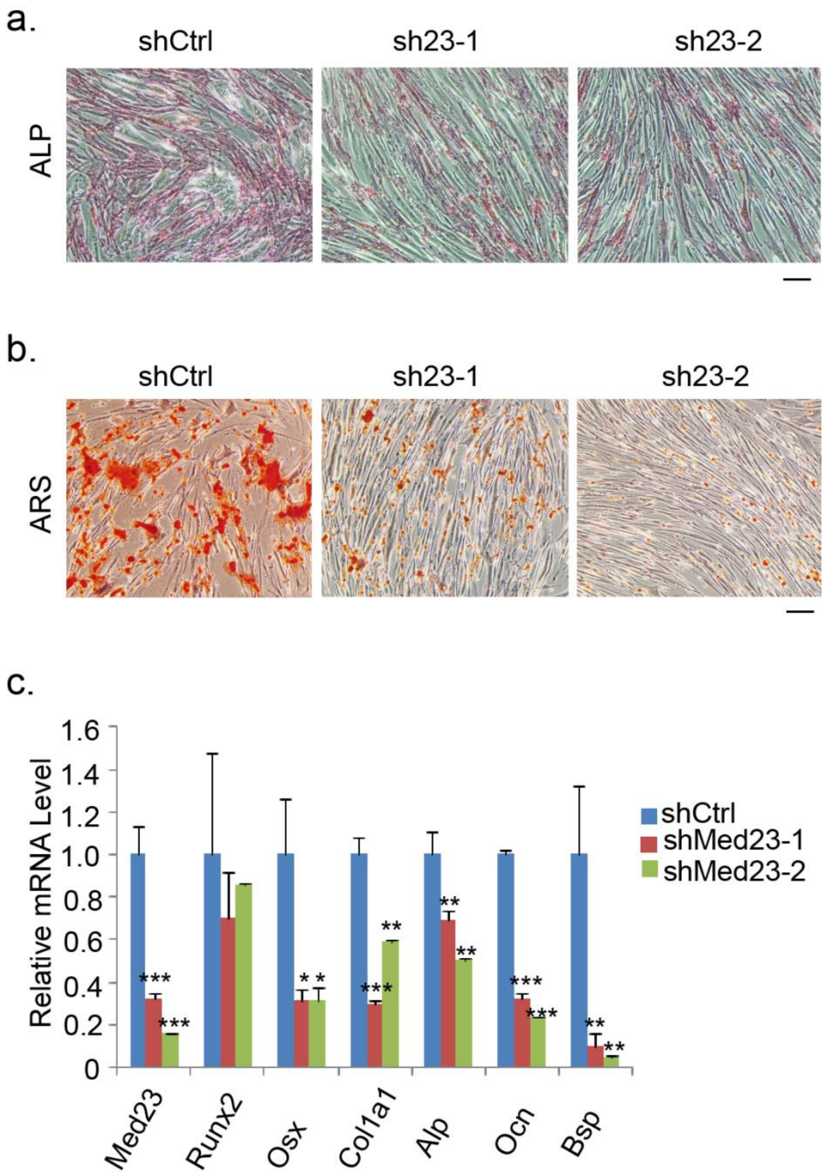

Supplementary Figure 9. The effect of Med23 deficiency on osteogenesis of primary human bone marrow-derived mesenchymal stem cells (hBMSCs) in vitro

(**a-b**) Primary hBMSCs were infected with retrovirus which expressed control or *hMed23* shRNAs, followed by cultured in osteogenic medium to differentiate into osteoblasts. ALP and ARS staining were performed at d7 (a) and d14 (b) respectively. Scale bar=100μm. (c) Expression of osteoblastic genes by real time PCR analysis. Data represent means ± s.d. (n=3 for each group, *t*-test).

Supplementary Figure 10.

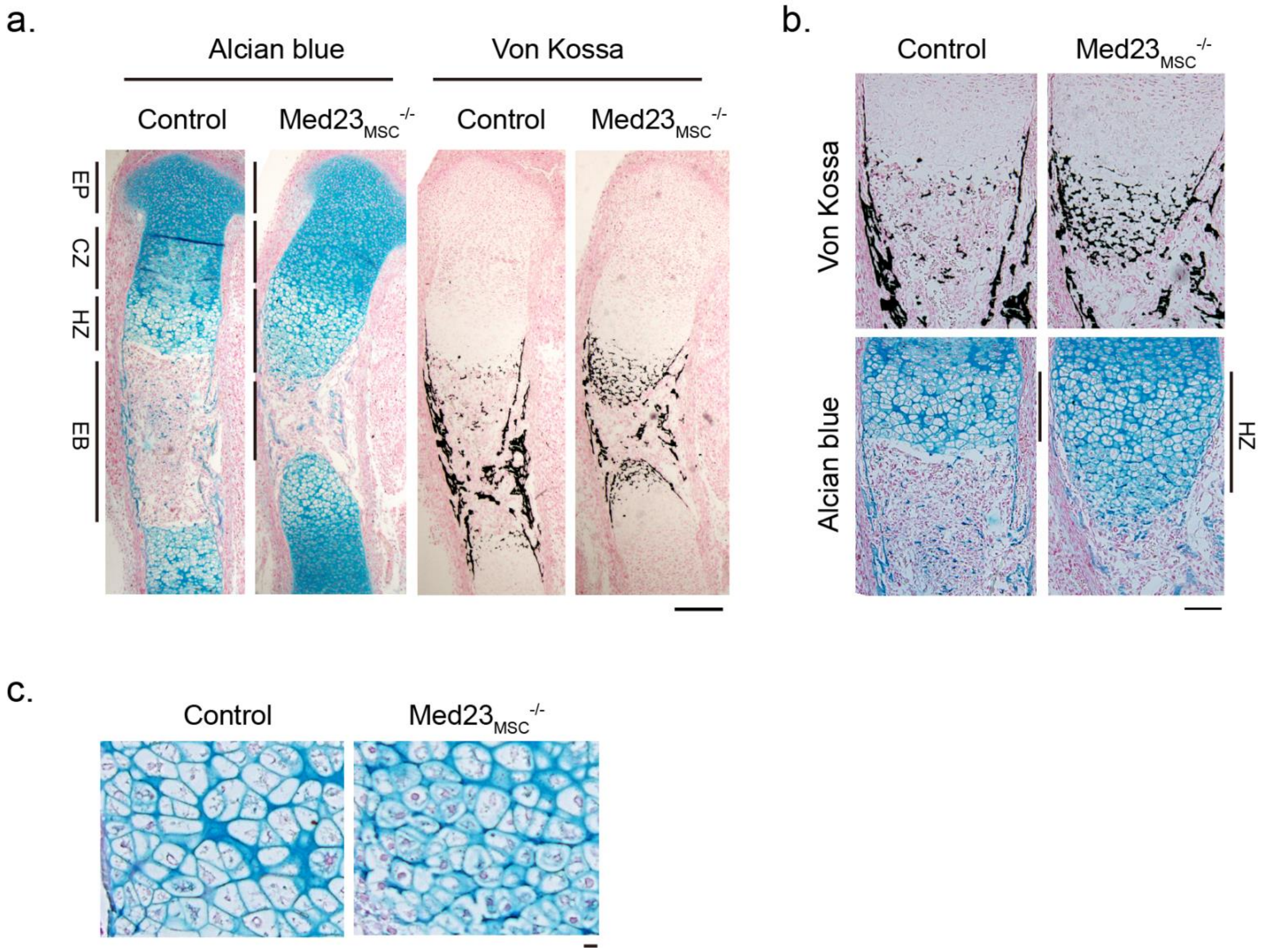

Supplementary Figure 10. Terminal maturation of chondrocytes are arrested in Med23-deficient mice (**a**). Histological analysis of tibia from E16.5 control (*Med23*$^{fl/fl}$) and *Med23*$_{MSC}^{-/-}$ mice by Alcian blue staining and Von Kossa staining according to standard protocols. EP, epiphyses zone; CZ, columnar zone; HZ, hypertrophic zone; EB, hypertrophic zone. Scale bar= 200 μm. (**b**). Zoomed-in image of HZ zone. Scale bar= 100 μm. (**c**) Zoomed-in image of hypertrophic chrondrocytes. Scale bar= 10 μm.

Supplementary Figure 11.

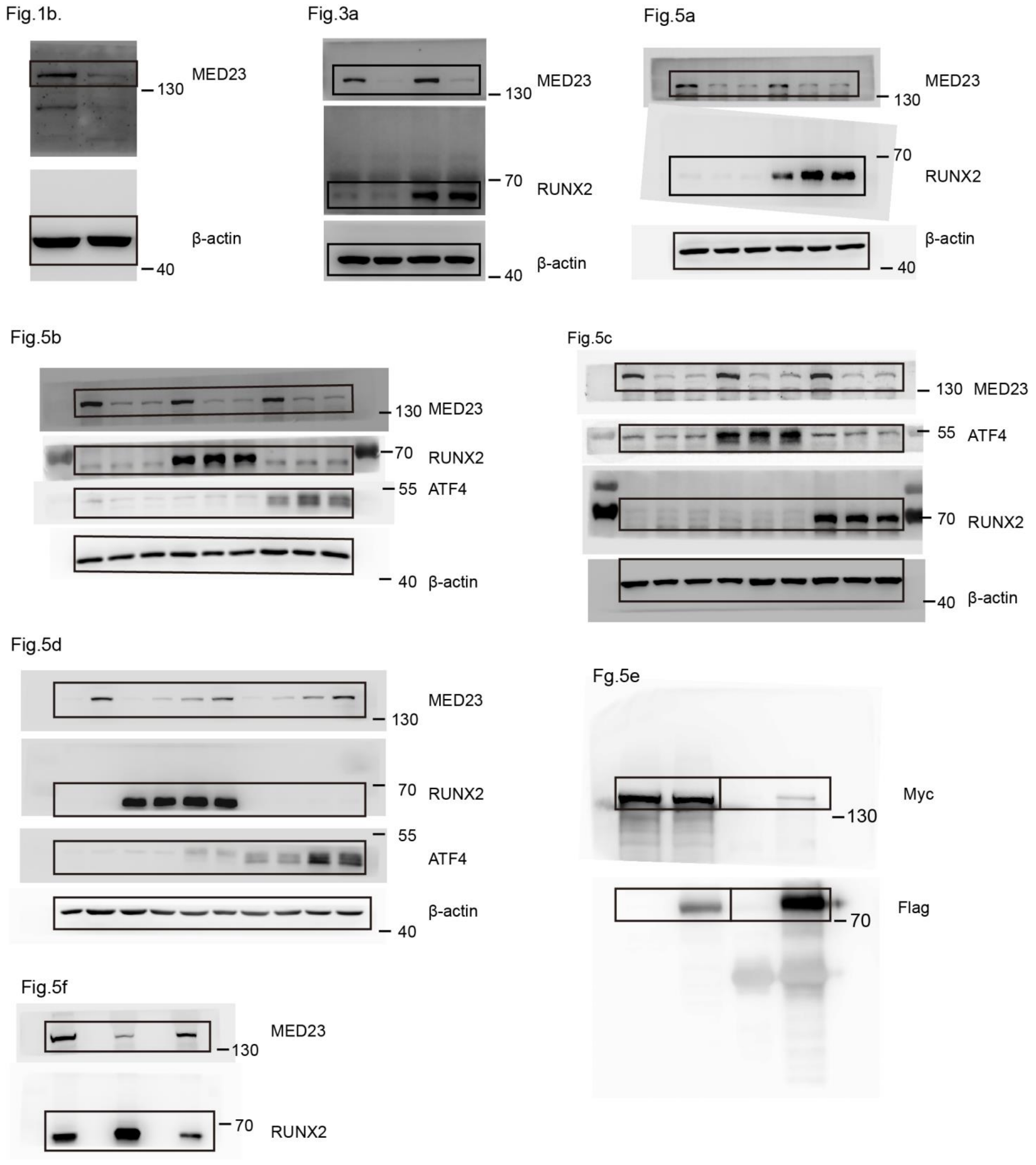

Supplementary Fig. 1c

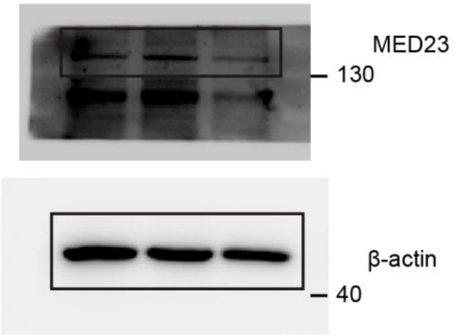

Supplementary Fig. 3b

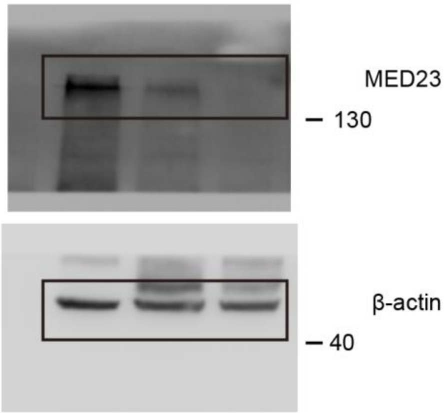

Supplementary Fig. 7a

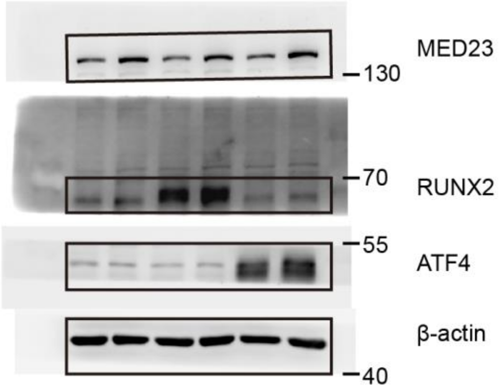

Supplementary Fig. 7b

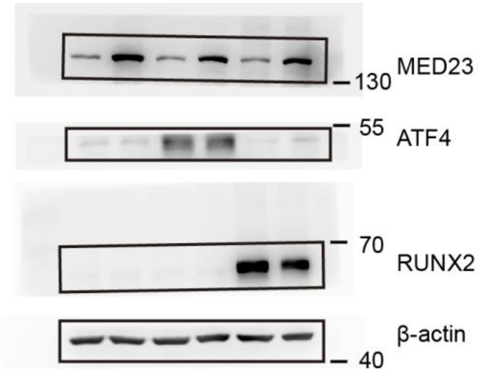

Supplementary Fig. 8a

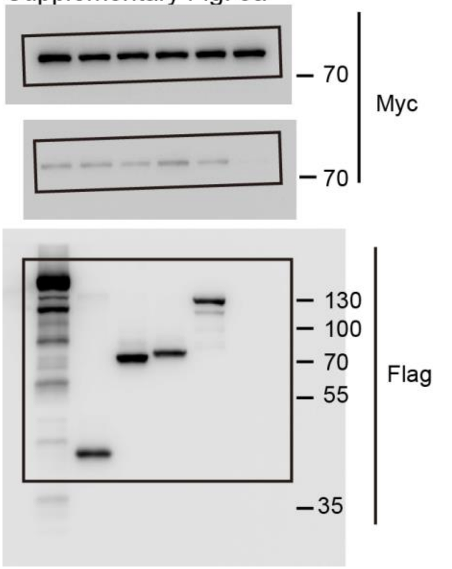

Supplementary Fig. 8b

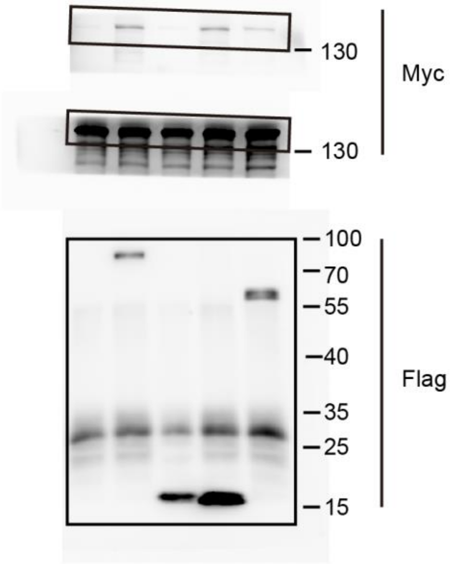

Supplementary Fig. 8c

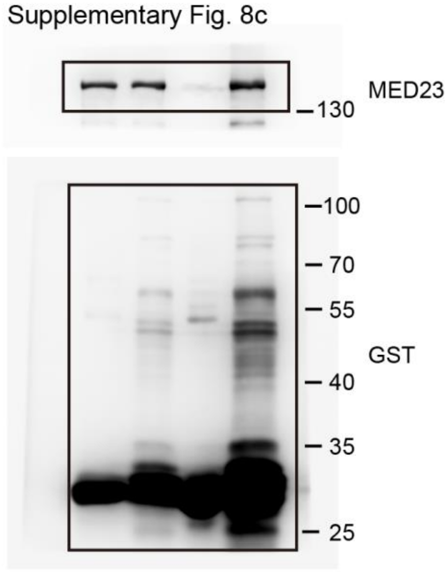

Supplementary Figure 11. Uncropped western blots for figures 1, 3, 5, and supplementary figures 1, 3, 7 and 8. The original immunoblots by SDS-PAGE gels cropped for main and supplementary figures are shown. Frames indicate shown bands in corresponding figures.

Supplementary Table 1. List of primer sequences for Quantitative RT-PCR

Gene symbol	Sequences (5' to 3')	
	Forward primer	Reverse primer
Gene expression, mouse genes		
Gapdh	AACTTTGGCATTGTGGAAGG	ACACATTGGGGGTAGGAACA
Med23	TCGGAAAATCATTGGAGGAG	CAATAGGCAGGCATTTCGTT
Runx2	CCCAGCCACCTTTACCTACA	TATGGAGTGCTGCTGGTCTG
Col1a1	GAGCGGAGAGTACTGGATCG	GTTAGGGCTGATGTACCAGT
Atf4	ATGACCGAGATGAGCTTCCT	TTACGGAACTCTCTTCTTCC
Osx	AGCGACCACTTGAGCAAACAT	GCGGCTGATTGGCTTCTTCT
Ocn	GCAATAAGGTAGTGAACAGACTCC	GTTTGTAGGCGGTCTTCAAGC
Alp	ATCTTTGGTCTGGCTCCCATG	TTTCCCGTTCACCGTCCAC
Bsp	GGGAGGCAGTGACTCTTCAG	CCCGAGAGTGTGGAAAGTGT
Ctsk	GAAGAAGACTCACCAGAAGCAG	TCCAGGTTATGGGCAGAGATT
Mmp9	AAAACCTCCAACCTCACGGA	GTGGTGTTCGAATGGCCTTT
Nfatc1	GACCCGGAGTTCGACTTCG	TGACACTAGGGGACACATAACTG
Oscar	CCTAGCCTCATACCCCCAG	CGTTGATCCCAGGAGTCACAA
Dcstamp	GGGGACTTATGTGTTTCCACG	ACAAAGCAACAGACTCCCAAAT
Src	GAACCCGAGAGGGACCTTC	GAGGCAGTAGGCACCTTTTGT
Gene expression, human genes		
GAPDH	CCAGAACATCATCCCTGCCT	CCTGCTTCACCACCTTCTTG
MED23	CACACCCTGAGCTGTTTTCC	GGAGCCCTGCATAGAGAAGT
RUNX2	GGCAGTTCCCAAGCATTTCA	AGGTGTGGTAGTGAGTGGTG
OSX	CCCTCCCTTTTCCCACTCAT	GGGTGTGTCATGTCCAGAGA
OCN	GCGCTACCTGTATCAATGGC	TCAGCCAACTCGTCACAGTC
ALP	CCCTTCACTGCCATCCTGTA	GCCTGGTAGTTGTTGTGAGC
BSP	CGATTTCCAGTTCAGGGCAG	CCTCTCCATAGCCCAGTGTT
COL1A1	ATGTGCCACTCTGACTGGAA	CTTGTCCTTGGGGTTCTTGC
ChIP assay, mouse gene		
Promoter region for Runx2 binding	GGCTGAGAGAGAGAGAGCAC	ATGTAAGCAGGAGGAGCAGG
Promoter region for Pol II binding	GAGAGCACAGAGTAGCCGAT	GAGTGAGCAGAGAGAGGGTC
Coding region	CAGTATGGCTTGAAGACCGC	AGAGAGAGAGGACAGGGAGG

Supplementary Table 2. The sequences of siRNA oligonucleotides:

	Sequences (5’ to 3’)
shMed23b (mouse)	GAGATAAGTAAGTTACATG
shMed23c (mouse)	GCAGCACGCACTTAAATTA
shMed23-1(human)	GGCAGCAAGAGAGGTTATA
shMed23-2 (human)	GAACCGAGCCTTGATATTG
shCtrl	GTGCGCTGCTGGTGCCAAC
